# Multifunctional Heteropentalenes: From Synthesis to Optoelectronic Applications

**DOI:** 10.1021/jacsau.2c00147

**Published:** 2022-05-10

**Authors:** Sebastian Stecko, Daniel T. Gryko

**Affiliations:** Institute of Organic Chemistry, Polish Academy of Sciences, Kasprzaka 44-52, 01-224 Warsaw, Poland

**Keywords:** heteropentalenes, optoelectronics, semiconductors, organic photovoltaics, organic field-effect transistors, pyrrolo[3,2-*b*]pyrroles

## Abstract

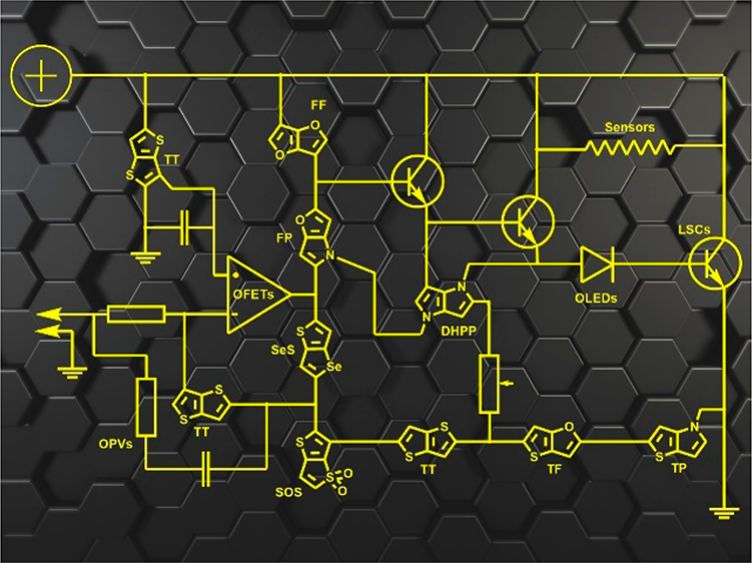

In the broad family of heteropentalenes, the combination of two five-membered heterocyclic rings fused in the [3,2-*b*] mode has attracted the most significant attention. The relatively straightforward access to these structures, being a consequence of the advances in the last two decades, combined with their physicochemical properties which match the requirements associated with many applications has led to an explosion of applied research. In this Perspective, we will discuss the recent progress of heteropentalenes’ usefulness as an active element of organic light-emitting diodes and organic field-effect transistors. Among the myriad of possible combinations for the different heteroatoms, thieno[3,2-*b*]thiophenes and 1,4-dihydropyrrolo[3,2-*b*]pyrroles are subject to the most intense studies. Together they comprise a potent optoelectronics tool resulting from the combination of appreciable photophysical properties, chemical reactivity, and straightforward synthesis.

## Introduction

**Figure 1:**
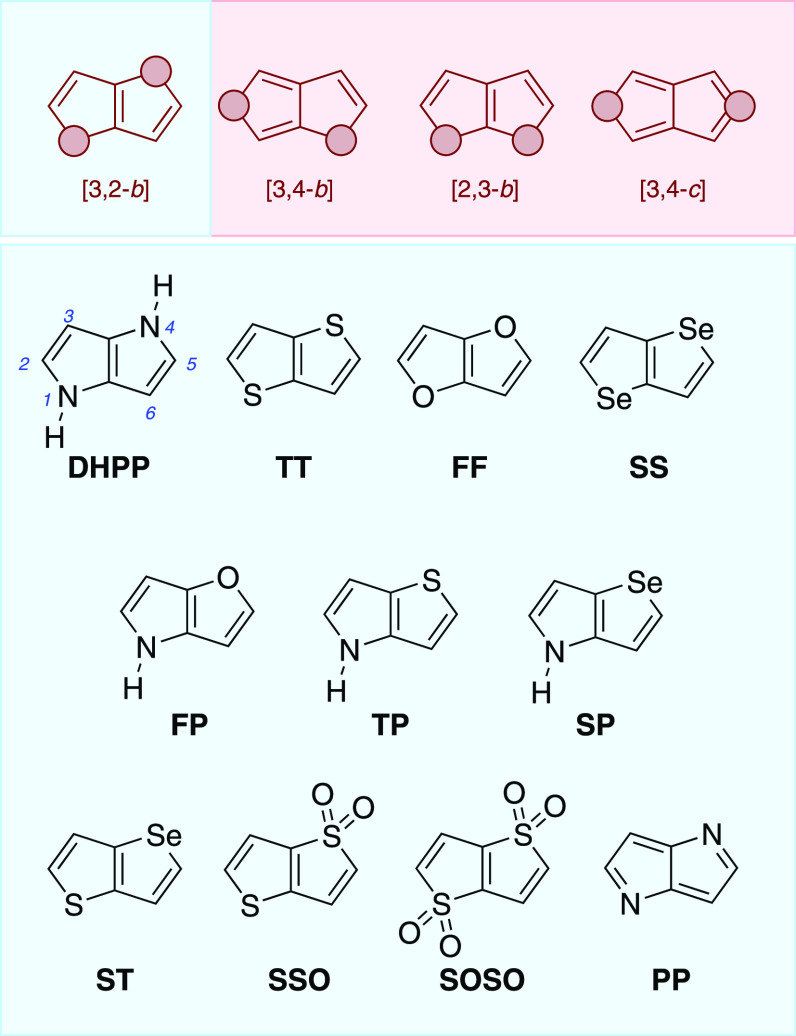
Structures of heteropentalenes.

Heteropentalenes (HPs) are a family of aromatic heterocycles composed of two fused five-membered rings.^1^ There are four distinct modes of fusion (Figure 1) and herein wee will focus only on the [3,2-*b*] pattern due to its importance reflected in its prevalence in the literature. Other regioisomeric HPs, e.g., [2,3-*b*], [2,3-*a*], and [3,4-*b*]^2^ (Figure 1), although very interesting, will not be described here due to space limitations. Pyrrolo[3,2-*b*]pyrrole (PP) structurally related to 1,4-dihydropyrrolo[3,2-*b*]pyrrole (DHPP) will not be included as well. Although [3,2-*b*]-fused HPs do not exist in nature, they have attracted significant attention during the last decades in relation to organic optoelectronics. There are at least three factors which distinguish [3,2-*b*]-heteropentalenes from both their structurally related regioisomers and indoles (or benzofurans): (1) Since they are typically built from electron-rich pyrrole, furan, and thiophene, they maintain their electron-rich character. Indeed, in the case of 1,4-dihydropyrrolo[3,2-*b*]pyrrole, the HOMO is located at −4.88 eV which makes it more electron-rich than pyrrole or indole.^3^ (2) They possess high molecular symmetry. This *C*_2*h*_ symmetry not only facilitates straightforward synthetic access to π-expanded analogues and makes them appealing building blocks in metal–organic framework construction, but also affects the molecular packing which in turn has a paramount importance in optoelectronic applications. (3) In general, their emission intensity is the strongest among regioisomeric HPs. The interest in [3,2-*b*]-HPs was enhanced with the advent of synthetic methods during the last two decades.

The Perspective will start with a short synthetic overview, followed by a description of general physicochemical and photophysical properties followed by a discussion of applied research in the area. It will not follow any chronological order, but it will be focused on the last 20 years. Although heteroatoms such as P, B, and Si were occasionally incorporated into HPs,^4^ we will focus entirely on O, N, S, and Se since they give rise to materials with the most promising optoelectronic properties. Undoubtedly, derivatives of thieno[3,2-*b*]thiophene (TT) and DHPPs are by far the most explored HPs, both as parent heterocycles and in the fused pattern, and the narrative will drift toward and focus on these two HPs. Unfortunately, due to space limitations, some interesting results could not be included in this Perspective including heterotetracenes^5^ and larger structures possessing more than two heteroatoms,^6^ for which we apologize to the respective authors.

## Synthesis

**Scheme 1:**
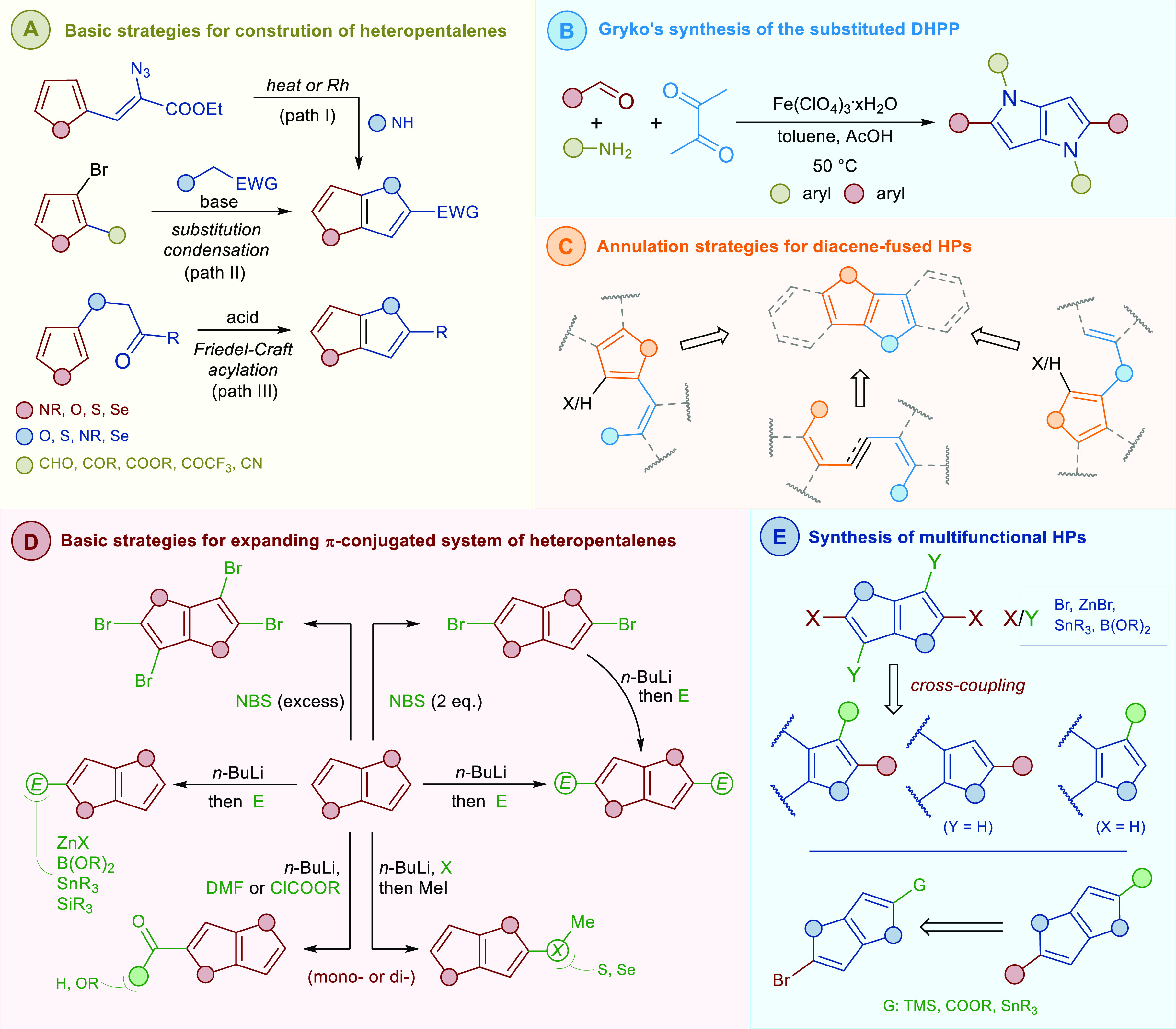
Summary of Synthetic Strategies Leading to Heteropentalenes

The synthetic methods leading to heteropentalenes could be divided in two major strategies depending on whether these heterocycles are π-expanded (Scheme 1A).^7^ In the case of parent systems, the most prevalent synthetic strategies rely on building the second five-membered ring fused to the one already present in the substrate’s structure. This is especially viable in the case of closing the pyrrole ring and thiophene ring.

This approach can be exemplified by the synthesis of 1,4-dihydropyrrolo[3,2-*b*]pyrroles (DHPPs) which was discovered in 1972 by Hemetsberger and Knittel.^8^ In general, this approach assumes the synthesis of an azido ester (via a Knoevenagel reaction of ethyl azidoacetate with the corresponding 1-formyl pyrrole derivative), which upon thermolysis subsequently cyclizes to provide the DHPP scaffold (Scheme 1A, path I). Depending on the application, the ester group at the 2-position can be later modified.^9^ As reported by Driver and co-workers,^10^ the thermolysis of the azide and C–H amination can be performed under milder conditions in the presence of rhodium catalyst.

This is a general method, and it allows the construction of a pyrrole ring from derivatives of thiophene, furan, or another five-membered ring, leading, respectively, to thieno[3,2-*b*]pyrroles (TPs) and furo[3,2-*b*]pyrroles (FPs).^7^

In an analogous manner, all other HPs can be prepared. For example, thieno[3,2-*b*]pyrrole (TP), discovered in 1957 by Snyder and Matteson, was synthesized from pyrrole by its transformation to a 3-thiocyano derivative, followed by the cyclization of the latter one and a subsequent reduction step by using NaBH_4_ (Scheme 1A, general path II).^11^ Another route to the same fused bicyclic system is through, as already recalled, Hemetsberger reaction of the corresponding thienyl azido acrylate, prepared via a Knoevenagel condensation reaction between thiophene-2-carboxaldehyde and azidoacetate. As already disclosed, the ester group at the 2-position can be later modified depending on the targeted application.^9^

Another common way to construct second heterocyclic ring of HPs is acid-promoted, intramolecular Friedel–Crafts acylation (Scheme 1A, path III).^12^

The above-mentioned methods are also suitable for the preparation of the multifunctionalized thieno[3,2-*b*]thiophenes (TTs).^12^ Another approach, particularly useful for the synthesis of symmetrically substituted TTs and benzo-fused TTs, relies on the simultaneous formation of both thienyl rings.^13^ In the same manner, the core of selenopheno[3,2-*b*]selenophene (SS) can be constructed.

The synthesis of π-expanded diacene-fused HPs, e.g., benzo[4,5]thieno[3,2-*b*]indole and benzo[*b*]benzo[4,5]selenopheno[2,3-*d*]thiophene (Scheme 1C), more or less follows the same strategies as stated before and relies on the addition of another ring to the existing heterocyclic core (Scheme 1C, paths I^14^ and II^15^). This includes, for example, the synthesis of 5,10-dihydroindolo[3,2-*b*]indoles via a double Buchwald–Hartwig amination.^16^ The third method, dedicated for the preparation of homoheteroatomic HPs (e.g., DHPP, TT, or SS), involves simultaneous formation of [3,2-*b*]-fused heterocyclic cores (Scheme 1C, path III) as in the case for the synthesis of TTs.^13^ Various straightforward methodologies leading to derivatives and π-expanded analogues of benzothieno[3,2-*b*]benzothiophenes include the reaction of (dichloromethyl)benzene with elemental sulfur, fusion of 2-chlorobenzaldehyde with NaSH and oxidative fusion of stilbenes possessing two MeS groups.^15c^ An analogous reactions with KSeCN can give rise to benzo[*b*]benzo[4,5]selenopheno[2,3-*d*]selenophenes. Finally, oxidation of benzothieno[3,2-*b*]benzothiophenes with *m*-CPBA or oxone leads to benzo[*b*]benzo[4,5]thieno[2,3-*d*]thiophene-5,5,10,10-tetraoxides.

In the field of the most electron-rich heteropentalenes, i.e., DHPPs, the real game-changer was the discovery of a multicomponent reaction of primary aromatic amines, aromatic aldehydes, and diacetyl that leads to tetraaryldihydropyrrolo[3,2-*b*]pyrroles (TAPPs).^17^ After a decade-long optimization, iron(III) perchlorate was identified as the best catalyst in this reaction (Scheme 1B).^18^ Under these conditions, a variety of aromatic aldehydes including derivatives of pyridine, pyrrole, thiophene, and furan, electron-rich and electron-deficient benzaldehydes, as well as sterically hindered aldehydes can be transformed into centrosymmetric TAPPs in yields reaching 77%. Over the past decade, more than 100 various TAPPs were synthesized following this general strategy and we have also proven that this reaction can be scaled up to 16 g.^19^

**Figure 2:**
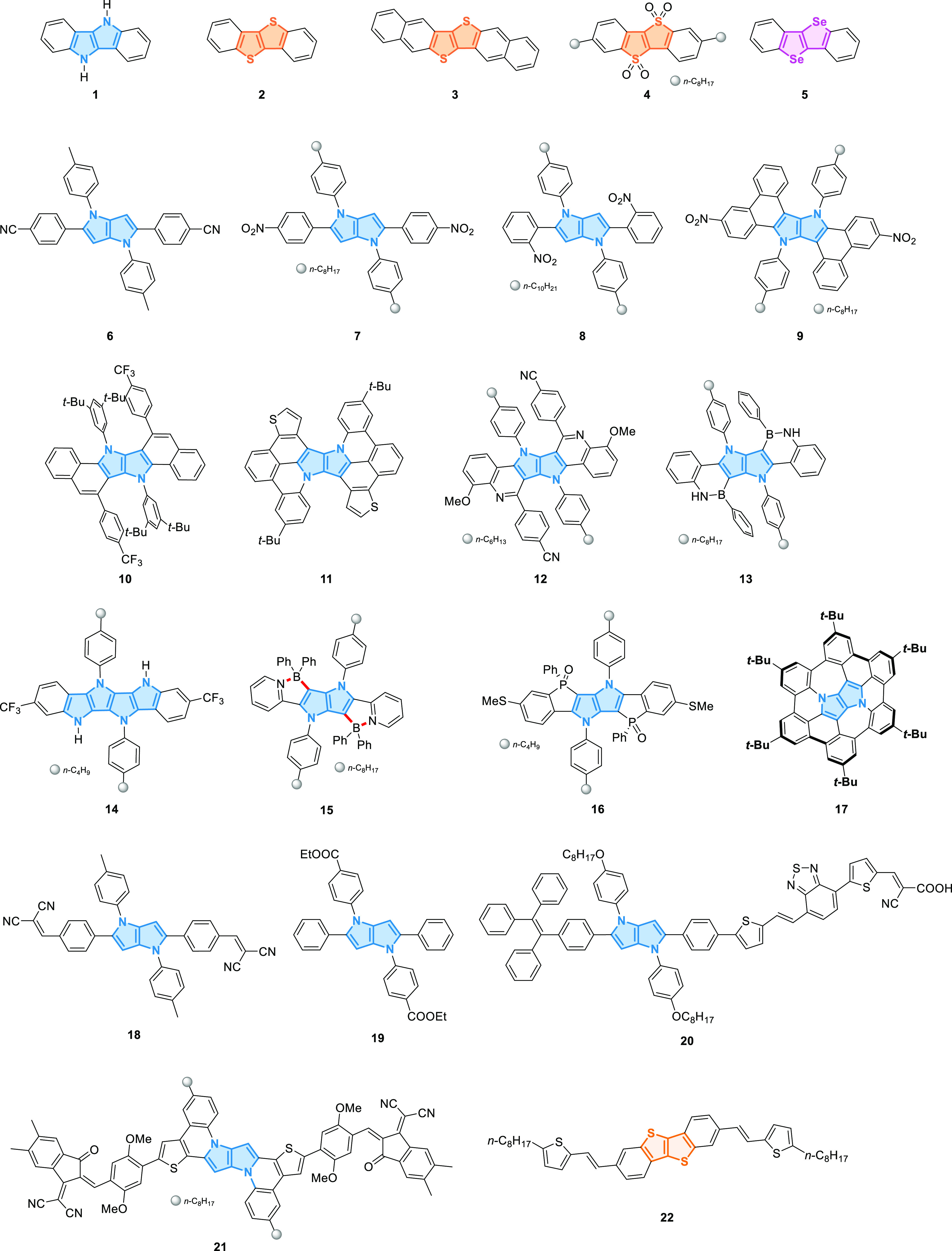
Structures of key π-expanded heteropentalenes, exemplary TAPPs, π-expanded DHPPs, and TTs.

The potential of this method cannot be underestimated. Since functional group compatibility is superb, the reaction gives rise to DHPPs possessing synthetic handles ready for further transformations. Importantly, all required substituents are installed at once around the periphery. The combination of this fact with the presence of highly reactive, electron-rich positions 3 and 6 prone to electrophilic aromatic substitutions opens a plethora of structural opportunities (**9**–**17**, Figure 2).^20^ In particular, this was exploited via various ring-closing reactions. It even turned out to be possible to connect all six substituents present at positions 1, 2, 3, 4, 5, and 6, creating an oval-shaped π-expanded DHPP **17** possessing a bowl shape.^21^

As summarized in Scheme 1D, the reactivity of HPs is analogous to that of the parent electron-rich heterocycles, i.e., pyrrole, furan, and thiophene. From a synthetic point of view, the bromination reaction is particularly useful, and, depending on the reaction conditions and applied reagents (Br_2_ or NBS), leads to either 2-bromo-, 2,5-dibromo-, or 2,3,4,5-tetrabromo-derivatives. In a similar manner, Friedel–Craft alkylation/acylation and Vilsmeier reactions can occur. The Friedel–Craft alkylation is particularly useful in the case of expanding the π-system via ladderization involving the HP backbone (vide supra). As for monocyclic congeners, an aromatic electrophilic substitution at positions 3 and 6 is less preferred; however, temporal deactivation of positions 2 and 5, for instance, by lithiation/silylation, allows for their functionalization as well.

Due to their stability, thieno[3,2-*b*]thiophene and its derivatives (e.g., 2-carboxylic acid) are commercially available. As a result, contrary to DHPPs, furo[3,2-*b*]pyrrole (FPs), or thieno[3,2-*b*]pyrrole (TPs), the common approach to afford multisubstituted TTs relies on simple functionalization of commercially available precursors to provide suitable building blocks for further transformations leading to complex π-extended molecular systems.

The deprotonation of HPs with organolithium reagents followed by quenching with an electrophile, e.g., RO-Bpin, TMSCl, and *n*-Bu_3_SnCl, is a common method for the preparation of 2-substituted or 2,5-disubstituted derivatives. The use of DMF or chloroformates, as electrophilic species, leads to formylated or alkoxycarbonylated derivatives, which are suitable precursors for copolimeric systems connected via a double bond.

Mono-, di- or tetrabrominated HPs as well as zinc, tin, or boron congeners are excellent substrates for Suzuki, Negishi, Stille, and Sonogashira cross-coupling reactions, enabling easy and rapid construction of structurally diversified π-extended molecular systems bearing a HP core. Moreover, as disclosed in Scheme 1E, depending on the location of the electrophilic functionalities, either a 2,5- or 3,6-substitution pattern can be achieved. Furthermore, the different reactivity of 2,5-dibromides and 3,6-dibromides allows for the regioselective formation of C–C bonds at C2 and C5, first, and then introduction of other functionalities at C3 and C6.

It is important to emphasize however that the strongly electron-donating character of DHPP cores affects the reactivity of the substituents either directly attached or linked through biaryl linkages to this scaffold. It was found that the reactivity of carbonyl groups and bromine atoms is greatly diminished so that some transformations such as the reduction of carbonyls or the Sonogashira reaction do not proceed at all.^22^

## General Physicochemical Properties

**Figure 3:**
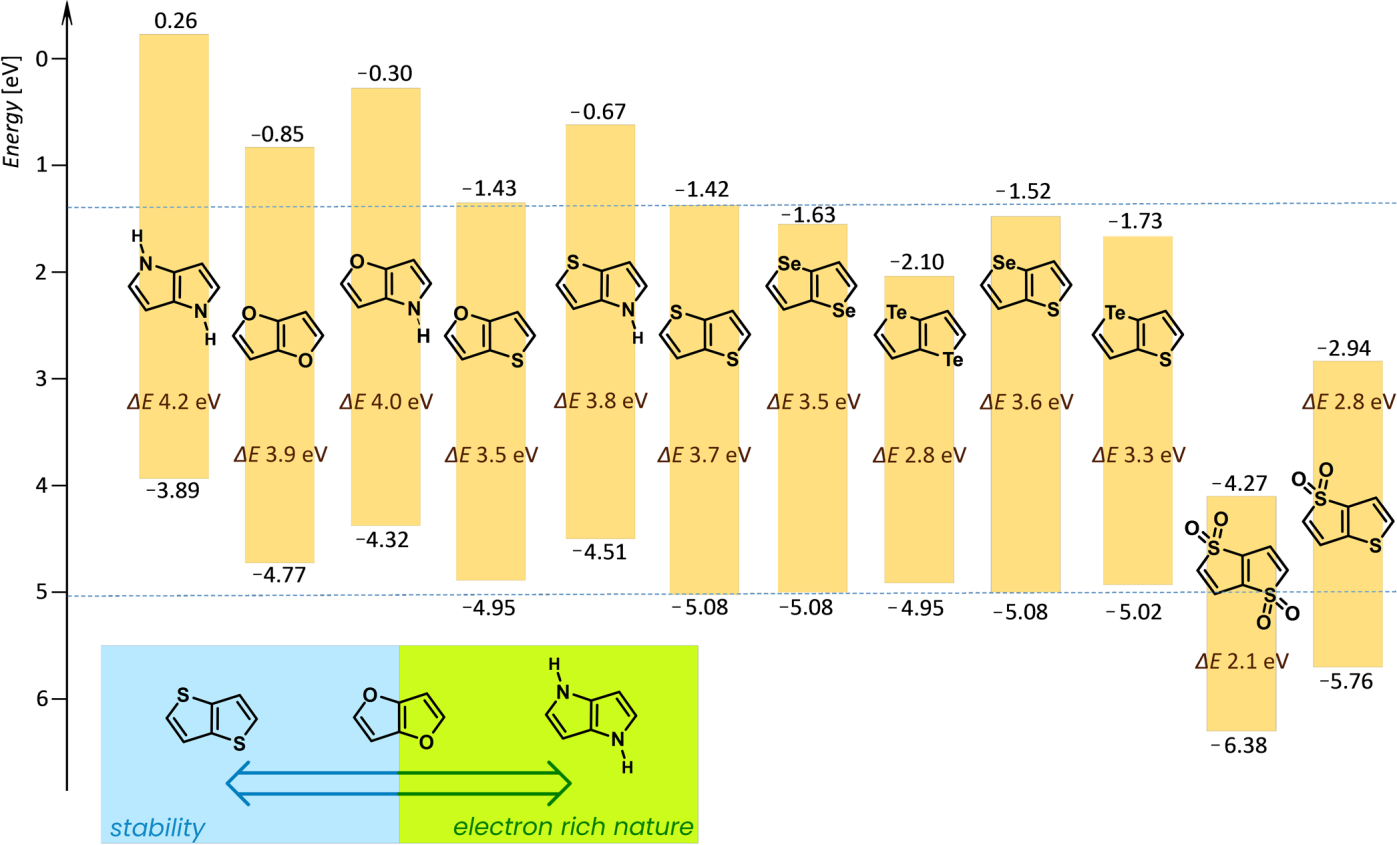
Calculated HOMO–LUMO energies of the various HP cores at the PBE0/6-31G(d) level (SDD was applied for Se and Te).^26^

The physicochemical properties of key heteropentalenes are strongly affected by the nature of the heteroatom(s),^23−25^ as exemplified by the energies of FMO, and energy of HOMO–LUMO gaps presented in Figure 3.^26^ In principle, their absorption maxima are located around 250 nm whereas emission was investigated only in several cases, e.g., λ_em_ (TT) = 291 nm and λ_em_ (PS) = 266 nm.^23b,27^ The key criteria that define efficient optoelectronic materials involve π-electron richness, aromaticity, electrical conductivity, dipole moments, frontier energy levels, thermal stability, and chemical stability.^28^ Due to the planarity and large charge carrier mobility, TTs have been widely used as electron-donating building blocks for organic photovoltaics (OPVs) (vide infra).^1b^ The key factor distinguishing thieno[3,2-*b*]thiophene from regioisomeric TTs is the enhanced propensity of forming a quinoidal structure. This difference combined with π-conjugation is directly responsible for the small optical gap, which translates to red-shifted absorption.

FF and DHPP are isoelectronic with TT and are likewise strong candidates for electronic materials. However, the systems constructed from these two scaffolds have garnered less attention even though these materials have demonstrated promising electronic properties, for instance, in both organic photovoltaics (OPVs) and organic field-effect transistors (OFETs).^29^ Of the previously mentioned fused five-membered aromatic rings, the electron richness decreases from pyrrole to furan to thiophene while stability trends in the opposite direction, as shown in Figure 3. The DFT calculations predict a high-lying HOMO level for pyrrole compared to furan and thiophene, indicating a relative instability toward atmospheric oxygen. The same trend can be found in the case of fused bicyclic congeners shown in Figure 3.^24,25^

In general, the poor air stability of pyrroles is considered to be a serious limitation when investigating and exploiting the electronic properties of pyrrole-derived materials. Thus, among the various heteropentalenes presented in Figure 1, DHPPs are the least studied system due to the disclosed π-electron excessive nature that leads to an unstable material. To circumvent this issue, electron-withdrawing groups can be placed adjacent to pyrrolic units. Another option would be to install electron-withdrawing units directly on the core while taking precautions to not perturb coplanarity.

## Photophysical Properties

As mentioned above, nonexpanded HPs have both absorption and emission located deep in the UV region. The ladder-type character of π-expanded heteropentalenes determines their photophysical properties to a large extent (Figure 2, Table 1). Indolo[3,2-*b*]indole (**1**) and dibenzothieno[3,2-*b*]thiophene (**2**) have an absorption still in the ultraviolet region of the electromagnetic spectrum and a rather weak emission. The same is true for benzo[*b*]benzo[4,5]selenopheno[2,3-*d*]selenophene (**5**). Interestingly, the oxidation of dye **2** to benzo[*b*]benzo[4,5]thieno[2,3-*d*]thiophene-5,5,10,10-tetraoxide (**4**) drastically changes the photophysical properties. Dye **4** becomes strongly emissive with a large Stokes shift originating from bathochromically shifted emission.^30^

**Table 1:** Photophysical Properties of Exemplary π-Expanded Heteropentalenes

compd	λ_abs_ (nm)	λ_em_ (nm)	Φ_fl_	ref
**1**^a^	360	400	nd^e^	ref (32)
**2**^a^	329	356	nd	refs (25), (33)
**3**^a^	403	nd	nd	refs (15b), (34)
**4**^a^	380	490	0.95	ref (30)
**5**^a^	344	nd	nd	refs (11), (22)
**6**^a^	406	461	0.24	ref (17)
**7**^b^	465	552	0.70	ref (35)
**8**^b^	428	nd	nd	ref (35)
**9**^b^	480	518	0.72	ref (36)
**10**^b^	379	465	0.26	ref (37)
**11**^a^	429	486	0.13	ref (20a)
**12**^b^	322	504	0.13	ref (20b)
**13**^b^	387	417	0.70	ref (38)
**14**^b^	406	419	0.82	ref (39)
**15**^b^	502	521	0.78	ref (20c)
**16**^a^	439	513	0.49	ref (40)
**17**^a^	530	615	0.016	ref (21)
**18**^a^	545	655	nd	ref (41)
**19**^c^	322	488	0.01	ref (42)
**20**^a^	548	nd	nd	ref (43)
**22**^d^	403, 425	444, 468	nd	ref (44)

a Absorption and fluorescence in CH_2_Cl_2_.

b Absorption and fluorescence in toluene.

c Absorption and fluorescence in THF.

d Absorption and fluorescence in CHCl_3_.

e nd: not determined.

One of the heavily studied phenomena of 1,4-dihydropyrrolo[3,2-*b*]pyrroles is the particularly strong electronic communication effect through positions 2 and 5. In the case of phenyl substituents, the corresponding dihedral angles are ca. 35° in the ground state and ∼25° in the S_1_ state.^2^^18,22a^ The combination of this fact and the strongly electron-rich character of pyrrolo[3,2-*b*]pyrrole core leads to the following: if substituents at positions 2 and 5 are strongly electron-withdrawing, the centrosymmetric, quadrupolar systems are created with markedly bathochromically shifted absorption and emission. Recent studies have revealed that if particularly strong electron-withdrawing groups are present, the emission maxima of TAPPs reaches 600 nm.^31^

In the case of π-expanded DHPPs, the range of skeletons studied during the past decade is overwhelming which reflects the availability of the TAPPs possessing suitable synthetic handles. The fusion of parent chromophores with additional moieties such as indole (**14**), quinoline (**12**), and phosphoxole (**16**) leads to skeletons possessing 8, 10, 12, or even 14 conjugated rings with planar or curved geometry. In most cases, the λ_abs_ is located at approximately 400–500 nm and emission is moderately bathochromically shifted which leads to rather small Stokes shifts (Table 1). Emission intensity depends on the particular heteroatoms involved in N-doping. The general trend is that with carbon-only analogues the Φ_fl_ is in the range of 0.1–0.2 whereas it is considerably stronger when a B–N bond is present as an isostere of C=C (dye **13**) and even strong when N → B dative bonds are present (structure **15**).

The extensive photophysical studies on TAPPs led to two important discoveries. TAPP **7**, possessing two 4-nitrophenyl substituents at positions 2 and 5, has almost quantitative fluorescence quantum yield in cyclohexane.^45^ This observation initiated extensive studies which revealed the crucial importance of the molecular geometry of the nitroaromatics for making them fluoresce. The planarity of the structures (TAPP **9**) ensures a spatial overlap between the orbitals carrying the positive charge of the oxidized donor (pyrrolopyrrolo core) and the negative charge of the reduced acceptor (nitrophenyl substituent) even for polarized CT states. Such orbital overlap, indeed, translates to large radiative decay rates and strong fluorescence, while the CT character of the excited states reduces the propensity for intersystem crossing (ISC) leading to triplet formation. Torsional degrees of freedom allow conformations with orthogonality between the rings of the donor and the acceptor breaking the delocalization of the frontier orbitals and diminishing orbital overlap. Therefore, such twisted intramolecular charge-transfer (TICT) excited states are dark. Furthermore, the orthogonal geometry of the TICT states enhances ISC. Solvent polarity stabilizes such TICT states with relatively well separated charges localized on the donor and the acceptor. The steric hindrance between the NO_2_ groups and the pyrrolopyrrole maintains orthogonality in the 2-nitrophenyl TAPP **8**, which is consistent with the lack of detectable fluorescence and the sub-picosecond excited-state lifetimes.^35^ These paradigms bring us closer to electron-deficient nitroaromatics that, in addition to their characteristics as n-type conjugates, also have attractive optical properties.^46^

The second disclosure originated from the extensive study of excited-state symmetry-breaking (ES-SB) by Vauthey and co-workers, focused predominantly on TAPP **6**.^47^ Detailed understanding of this photophysical phenomenon requires the ability to monitor it in real time. The monitoring of the CN stretching modes on the acceptor units using ultrafast time-resolved infrared spectroscopy enables ES-SB to be visualized. The presence of two CN bands in polar solvents points to ES-SB occurring within ca. 100 fs. On the contrary, in apolar solvents, the S_1_ state remains symmetric and quadrupolar as shown by the presence of one band. In protic solvents, H-bonding interactions significantly amplify ES-SB. These studies led Ivanov to propose the new model for ES-SB.^48^

Interestingly, some TAPPs possessing electron-withdrawing groups attached to positions 1 and 4 display aggregation-induced emission (AIE).^42,49^ The authors claim that the restricted intramolecular rotation (RIR) is the key mechanism responsible for the AIE character displayed by TAPP **19**. It was also found that the temperature and solvent polarity modulate both RIR and TICT processes in **19**. The strong polarization of A-D-A type TAPPs is reflected also in their relatively large two-photon absorption cross-section (σ_2_). Given that TAPP **6** contains multiple biaryl linkages, σ_2_ = 600 GM represents one the highest values in this type of dyes.^45^

## Optoelectronic Applications

A vast number of HP derivatives, particularly TPs, FPs, as well as TTs, have found application as medicines or pesticides.^50^ However, their key applications are in the electronic domain of conducting polymers, liquid and clathrate crystals, organic conductors or superconductors, photosensitive receptors, materials for optoelectronics (nonlinear optical chromophores), and dyes.^51^

The backbone conformation of the conjugated polymer backbones directly influences the electronic coupling which determines the optoelectronic properties.

The charge transfer is mainly composed of an intrachain and an interchain charge transport. The first mode is directly determined by the π-orbital overlap along the condensed ring backbone.^52,53^ The rigid coplanar conformation of the conjugated molecular system backbone could promote, which are advantageous to.

There are two key factors making a rigid coplanar backbone to be crucial to the efficient charge transfer of π-expanded molecular systems. Predominantly the intrachain charge transfer is accelerated as a result of the π-orbital coupling, leading to long carrier delocalization length and low band gap. This is reinforced by facilitated interchain charge transport^54^ resulting from strong π–π interactions between polymers chains which also originates from the extended π-plane.

To improve the rigidity and coplanarity of π-expanded molecular systems, considerable strategies have been developed based on molecular design. According to the linking type between the building blocks, these rigid coplanar molecular systems can be divided into three categories: (1) noncovalent interactions locked materials, (2) double-bond linked materials via quinonoid form, instead of the free-rotational single-bonds linked building blocks, and (3) fully conjugated ladder-type molecular systems.

**Figure 4:**
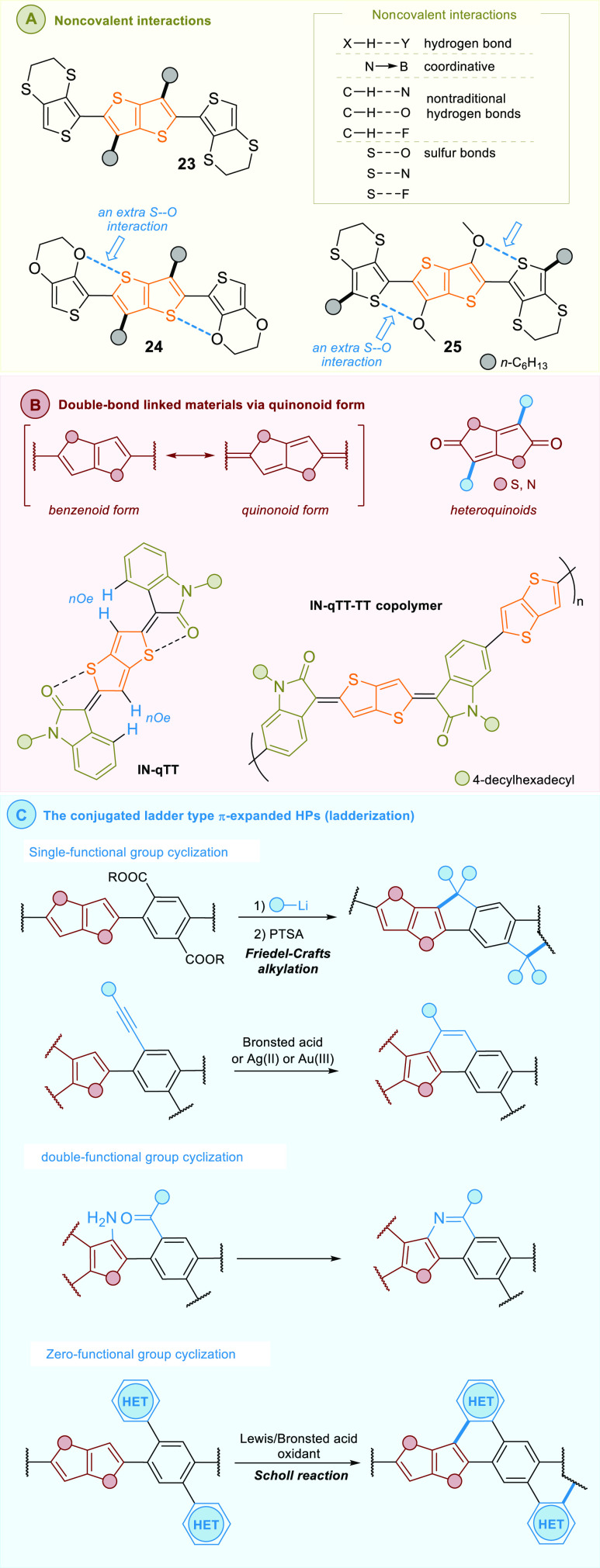
Conformation control of HPs.

The coplanar conformation can be enforced by introducing noncovalent interactions into the semiconducting molecular system.^55^ Noncovalent interactions (Figure 4A), including hydrogen bonds, coordinative interactions, and “sulfur bonds,” refer to the interactions between two functional groups bound solely by electronic multipole–electric multipole attraction and repulsion.

These approaches to provide planarity along molecular systems has been also applied for the π-expanded HP backbones. For example, electrochemical and optical investigations of TT-based conjugated molecules **23** and **24** reported by Skabara and co-workers,^56^ provided the repeating band gaps as 2.21 and 3.01 eV for **24** and 2.45 and 3.14 eV for **24**. As indicated, the reason was the twisted conformation observed for the 3,4-ethylenedithiothiophene in molecule **23**, while in the case of **24** noncovalent S···O interactions resulted in a planar conformation and hence a high degree of conjugation length (Figure 4A). Thus, the hole mobilities for **24** and **23** differed (4.0 and 1.5 × 10^–2^ cm^2^/(V s), respectively). A similar phenomenon was observed by Frère et al.,^57^ who investigated the properties of molecule **25**. Here, also the S···O intramolecular interactions along with a rigid TT unit led to a planar conformation. Moreover, Skabara et al.^58^ and others^54b^ unveiled that also the S···N intramolecular interactions can provide the molecule with a more rigid structure.

Semiconducting and optoelectronic materials bearing HP-based quinoidal building blocks provide rigidity and coplanarity to the molecular scaffold and are an emerging class of materials (Figure 4B).^59^ In contrast to their aromatic counterparts, quinoids show significant bond length alternations that break aromaticity and therefore reduce the HOMO–LUMO gap that potentially enables the injection of both electrons and holes. The different chemical reactivities and low-energy and low-symmetry spectroscopic features guarantee new functionalities. For small-molecule semiconductors, this structure enjoys widespread use in n-type materials because of the ease of electron injection leveraging the combined contribution of small energy gap and low-lying LUMO energy levels.

Typical examples are semiconducting polymers based on oxindole-capped quinoids. Reichmanis and co-workers^60^ prepared the quinoidal TT-based building block (IN-qTT) through a two-step sequence involving organolithium-mediated addition and SnCl_2_-mediated reduction. In contrast to thiophene and bithiophene counterparts, a quinoidal-TT spacer leverages its innate symmetric molecular geometry and reduced configurational disorder to build a polymer backbone. The centrosymmetric geometry of IN-qTT was confirmed by NOESY experiments. This compound was copolymerized with the TT monomer to introduce both quinoidal and aromatic substructures. Bond length alternation in the quinoidal structure is a typical feature proven by theoretical modeling. This alternation motif resulted in a lowered HOMO–LUMO gap and a different frontier orbital distribution in comparison with that of the isoindigo-based polymers. The thin film transistor based on the IN-qTT-TT copolymer showed a hole mobility of 0.13 cm^2^/(V s), which was slightly lower than that of the isoindigo-TT counterpart. This result implies a gap-narrowing strategy by the insertion of qTTs and expands the chemical space of thienoquinoid-embedded materials for organic electronics.

An extension of the quinoid form enhances rigidity and planarity of semiconductors and optoelectronics and is of use in heteroquinoids, such as pyrrolo[3,2-*b*]pyrrole-2,5-dione and thieno[3,2-*b*]thiophene-2,5-dione. The introduction of heteroatoms to quinoid frameworks polarizes the bonds for enhanced electronic effects and chromophoric matters. The unpaired electrons from nitrogen or sulfur atoms directly conjugate into the π-system to afford an isoelectronic structure similar to an aromatic sextet. These molecules can be substituted at the 3- and 6-positions to build a backbone, in which the conjugation pathway resembles a butadiene in a fused mode. The carbonyl groups offer moderate-to-high electron-accepting capacities, together with a polarized structure, to fine-tune the frontier orbital energies. In this context, these moieties are promising candidates for electron-acceptors in polymeric semiconductors.^59^

The third approach to provide rigidity and coplanarity of heteropentalenes is conjugated ladderization (Figure 4C),^55b^ the π-system is expanded via cyclization of the backbone to enhance π-orbital overlap along the entire molecule. The methods of cyclization can be divided into three groups: (1) double-functional-group cyclization, efficient cyclization between two preinstalled functional groups on neighboring aromatic rings (e.g., via olefin metathesis^61^ or Schiff base condensation;^62,20b^ (2) single-functional-group cyclization, mainly via electrophilic aromatic substitution with a preinstalled functional group to the neighboring aromatic ring, like Friedel–Crafts reaction^63,64^ or acid-mediated alkyne cyclization;^65,37^ (3) zero-functional-group cyclization, i.e., oxidative coupling between two neighboring aromatic rings via Scholl reaction.^66^

**Figure 5:**
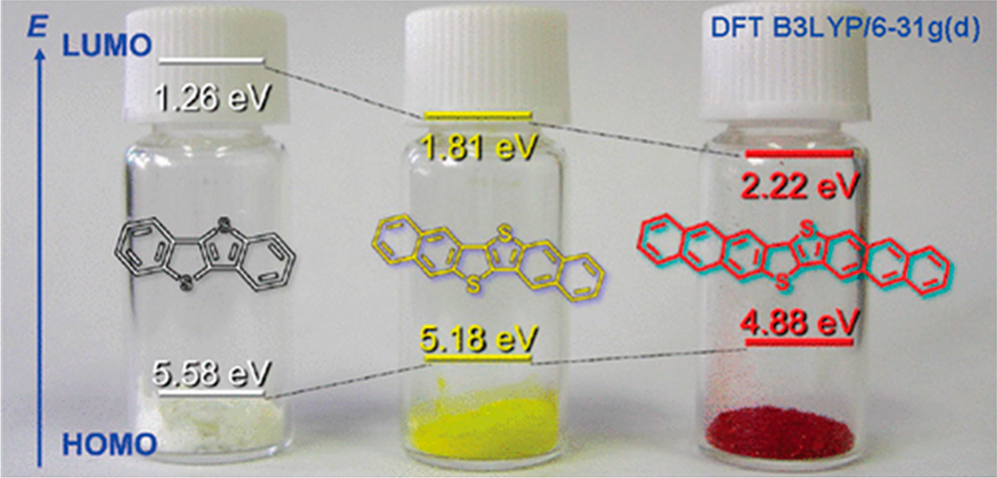
Calculated FMO energies of **2** (BTBT), **3** (DNTT), and DATT at the DFT B3LYP/6-31g(d) level of theory. Reproduced from ref (15e). Copyright 2011 American Chemical Society.

Due to their properties, multisubstituted TTs as well as their condensed analogues are key structural elements of dyes for optoelectronic purposes. An another way to reduce HOMO–LUMO gaps in heteropentalenes is to expand the π-system by adjusting additional aromatic rings to provide diacene-fused HPs. For example, Takimiya and co-workers reported the synthesis and evaluation of electronic properties of diacene-fussed TT, i.e., benzothieno[3,2-*b*]benzothiophene (BTBT, **3**),^67^ dinaphto[2,3-*b*:2′,3′-*f*]thieno[3,2-*b*]thiophene (DNTT, **4**),^15c,15d,68^ and dianthra[2,3-*b*:2′,3′-*f*] thieno[3,2-*b*]thiophene (DATT)^15c,15e^ (Figure 5). These π-extended molecular frameworks are quite useful for the development of high-performance OFETs and their integrated devices and circuits.^69^ As shown in Figure 5, an expansion of the π-system results in a decrease of the HOMO–LUMO gap. It also changes the absorption properties: BTBT is a white solid, whereas DNTT and DATT are yellow and red, respectively. As a result, the parent DNTT- and DATT-based OFETs show mobilities as high as 3.1 cm^2^/(V s) for thin film transistors^15c,15d,68^ and 8.3 cm^2^/(V s) on single crystals.^70^ Furthermore, alkylated DNTT derivatives show much enhanced mobilities in vapor-deposited thin film transistors (up to 7.9 cm^2^/(V s))^71^ and solution-processed single-crystalline film transistors (up to 12 cm^2^/(V s)).^71^ Notably, although the DNTT and DATT core has a highly π-extended acene structure consisting of six and eight fused aromatic rings, these molecules are very stable due to the TT moiety embedded in the middle of two naphthalenes or anthracenes.^71^ Geerts and co-workers^72^ found out that the molecular packing strongly influences two factors governing the charge transport, i.e., the ionization potential and transfer integrals. The highest interfacial mobility of 1.7 × 10^2^ cm^2^/(V s) was measured for 2,7-didodecyl-benzothieno[3,2-*b*]benzothiophene. Indeed, the molecular arrangement in a crystal lattice and the crystallinity are considered to be two key factors deciding the carrier mobility. Using a novel off-center spin-coating method, Bao and co-workers^73^ were able to form the highly aligned films from 2,7-dioctylbenzothieno[3,2-*b*]benzothiophene and polystyrene. This resulted in one of the highest thin film transistor hole mobilities for all organic molecules with 25 cm^2^/(V s).

**Figure 6:**
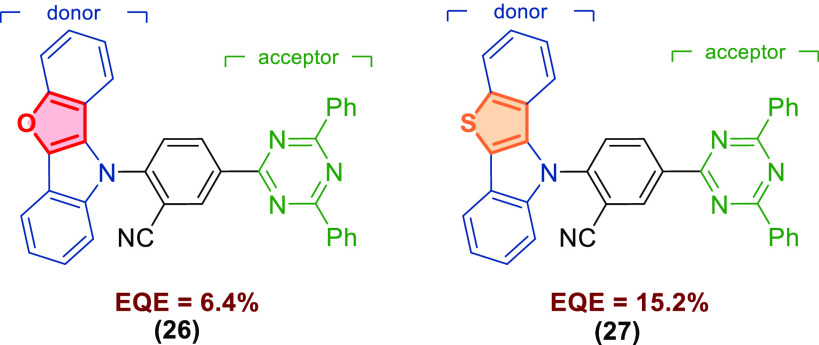
Effect of heteroatom (O or S) replacement in the donor scaffold on the physicochemical and electroluminescence properties of TDAF emitters.^14c^

The physicochemical and optoelectronic properties of HPs and their derivatives are also influenced by the type of heteroatoms present in the [3,2-*b*]-fused core. For example, Hong and co-workers^14c^ synthesized two green TADF emitters, **26** and **27**, bearing benzofuro[3,2-*b*]indole and benzo[4,5]thieno[3,2-*b*]indole, respectively, as donors and an aryltriazine acceptor and studied the effect of the heteroatom in the donor scaffold on the photophysical and electroluminescence properties (Figure 6). Due to the different electronegativity values, the replacement of O by S, caused the change of donor properties, which resulted in the modulation of a singlet–triplet energy splitting (Δ*E*_ST_) value and, as a consequence, thermally activated delayed fluorescence (TADF) mechanism of the compounds. The thiophene-based donor was advantageous in obtaining small Δ*E*_ST_ and high reverse intersystem crossing constant values (*K*_RISC_), a strong delayed fluorescence component, and superior upconversion efficiency when compared with its furan congener. Notably, small Δ*E*_ST_ values and efficient RISC are key parameters in the design of efficient TADF emitters. Therefore, the device containing **27** exhibited superior performance with an external quantum efficiency value of 15.2% that was more than 2 times higher than that for **26**.

**Figure 7:**
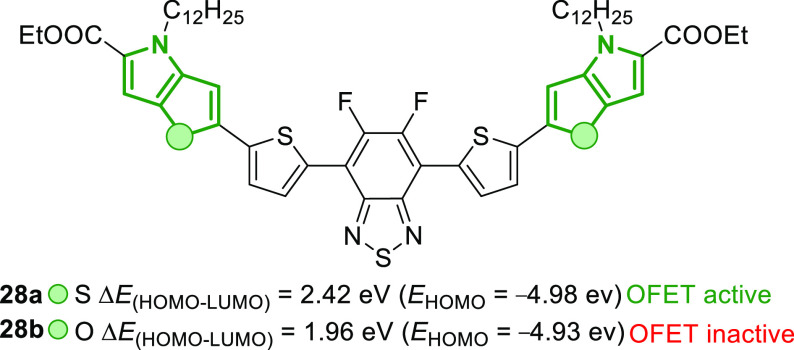
Effect of heteroatom (O or S) replacement in the donor scaffold on the OFET properties of D-A-D type semiconductors.^74^

Stefan and co-workers^74^ prepared D-A-D type organic semiconducting small molecules **28** and evaluated their activity in the organic field-effect transistor (OFET) performances (Figure 7). The FP containing molecule **28b** showed an enhanced absorption toward NIR I and NIR II regions. Both molecules had similar HOMO levels, but compound **28b** possessed a low lying LUMO level to realize a low band gap semiconductor. Molecule **28a**, containing TP units, exhibited moderate hole mobilities (10^–3^ cm^2^/(V s)) irrespective of the annealing temperature. In contrast, its congener **28b** bearing FP was completely inactive in field-effect transistors.

**Figure 8:**
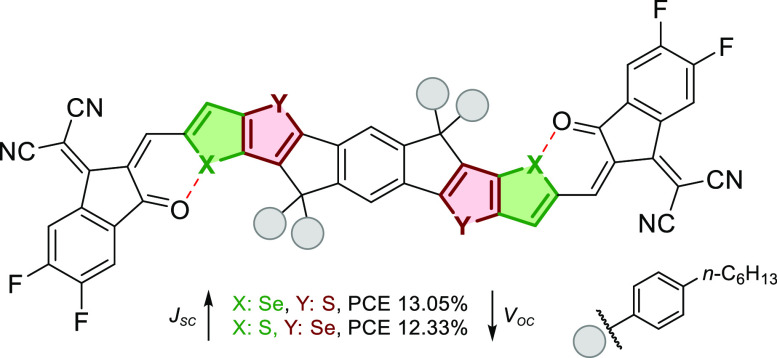
Influence of the location of the Se atom on PCE.^79^

The intermolecular Se–Se interactions are stronger than S–S ones, which is a key factor behind improving distinct solid-state organization and, therefore, charge carrier mobilities.^75^ There are two pivotal reasons for that. Firstly, since the lone pairs on selenium atoms do not contribute to the aromaticity of the π-conjugated system as much as the sulfur does, their interaction is stronger. Additionally, the aromaticity of selenophene is reduced which in turn narrows its band gap. This is related to its higher quinoid resonance character in the ground state which is a direct consequence of the looser electron cloud delocalization around the more polarizable selenium atom.^75,76^ Earlier, Liao and co-workers^63,77^ reported that the introduction of selenium into the molecular framework of nonfullerene acceptors (NFA) gave PCEs of >8%. The best performance to date for binary organic solar cells based on selenophene-incorporated NFAs was revealed by Hou et al. Incorporation of a selenopheno[3,2-*b*]thiophene unit into A-D-A type NFA resulted in PCE = 13.3%.^78^ Recently, Jen and co-workers^79^ showed that the specific location of Se atoms in NFA not only affects the optical and electrochemical properties but also changes the morphology of thin films. An explanation of this feature is the presence of an extra Se–O interaction that further stabilizes the system (Figure 8).

**Figure 9:**
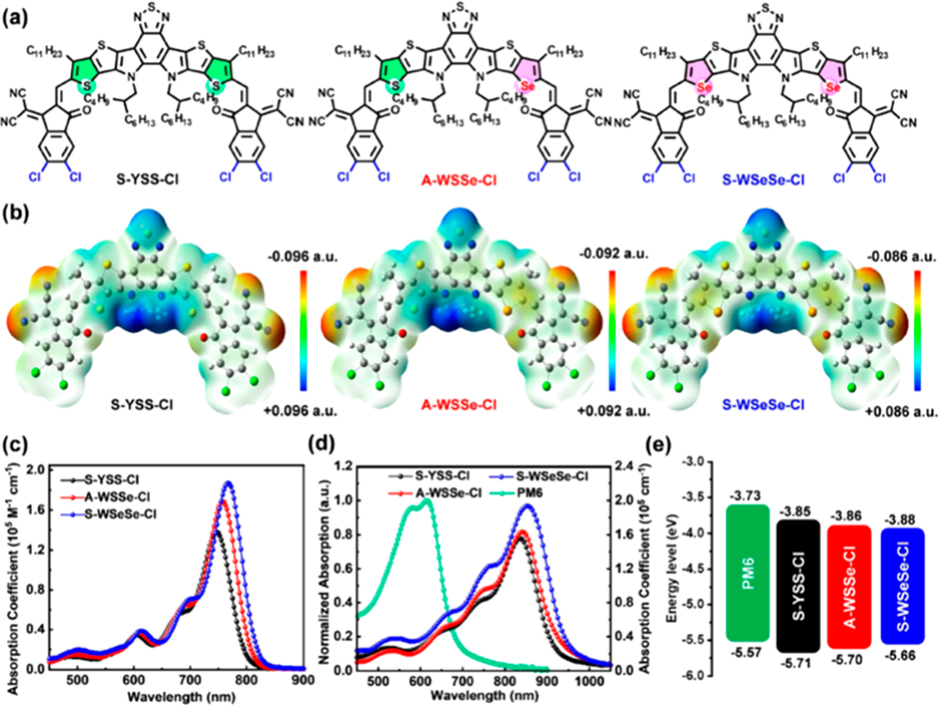
(a) Molecular structure and (b) corresponding electrostatic potential (ESP) distributions of S-YSS-Cl, A-WSSe-Cl, and S-WSeSe-Cl. (c) Molar absorption coefficient spectra of S-YSS-Cl, A-WSSe-Cl, and S-WSeSe-Cl in CHCl_3_ solutions. (d) Absorption spectra of PM6, S-YSS-Cl, A-WSSe-Cl, and S-WSeSe-Cl in drop-cast thin films. (e) Energy levels (calculated from CV) of PM6, S-YSS-Cl, A-WSSe-Cl, and S-WSeSe-Cl in drop-cast thin films. Reproduced with permission from ref (80). Copyright 2021 John Wiley and Sons.

Recently, Wang and co-workers^80^ reported the synthesis and physicochemical studies of symmetric or dissymmetric A-DA′D-A type nonfullerene small molecular acceptors bearing different numbers of selenophene units (Figure 9). The authors proposed a new strategy to improve *J*_sc_ and fill factor without sacrificing *V*_oc_ via the combination of a dissymmetric core and precise replacement of the selenophene on the central core. This approach led to a spectacular result, namely, a A-WSSe-Cl-based device with a PCE of 17.5%, which is the highest value for selenophene-based NF-SMAs in binary polymer solar cells. Of note, within the family of dyes, an increase in electron mobility and crystallinity in neat thin films was observed while moving from S-YSS-Cl to A-WSSe-Cl and to SWSeSe-Cl. Authors attributed the best performance of A-WSSe-Cl and S-WSeSe-Cl (compared to the thiophene-based S-YSS-Cl) to stronger and tighter intermolecular π–π stacking interactions as well as to an extra S···N noncovalent intermolecular interactions from the central benzothiadiazole. Another key factor which was identified is better ordered 3D interpenetrating charge-transfer networks.

In recent years, the promising class of glycolated semiconducting materials, including copolymers of TT, has dominated the field of organic electrochemical transistor (OECT) channel materials.^81^ Such glycol chain decorated semiconducting polymers mirror previous conjugated polyelectrolyte materials in that the electronic charge transport occurs along the π-conjugated backbone while ion transport is facilitated by the hydrophilic glycol side chains.^82^ Use of the thieno[3,2-*b*]thiophene unit as one of the monomers, instead of the commonly used bithiophene, leads to negligible changes in the optoelectronic properties of the resulting copolymers which however possess improved hole mobility due to the rigidity of the TT moiety causing shorter π–π stacking distances.^82a^

Dye-sensitized solar cells (DSSCs)^51^ have attracted much attention in the past couple of decades due to their ability to efficiently convert solar energy to electricity at a low cost being a promising solution for the energy crisis. Most of the metal-free organic sensitizers employed in DSSCs consist of an electron donor and an acceptor connected by a π-conjugated linker, thus presenting wide possibilities for structural modification.^83^ In such dye systems, triarylamine and cyanoacrylic acid, or another electron-withdrawing moiety, are widely employed as donor and acceptor units, respectively, and various π-conjugated linkers are used to bridge the donor and acceptor units to create a diverse range of D−π–A dyes for DSSCs.^83^ The π-spacer plays an important role in tuning the molecular band gap, while electronic and steric factors also have strong impacts on device performance. Due to their properties, multisubstituted heteropentalenes, as well as their condensed analogues are key structural elements of dyes for optoelectronic purposes.

One of the perspective devices toward near-zero energy consumption buildings are luminescent solar concentrators (LSCs). Beverina and co-workers^30^ proposed the utilization of π-expanded SOSO derivatives in this regard. Optical power efficiency as high as 3% was obtained with dye **4**, which corresponds to an optical quantum efficiency of 54%. The marked advantages of benzo[*b*]benzo[4,5]thieno[2,3-*d*]thiophene-5,5,10,10-tetraoxide **4** are its high thermal, chemical, and photochemical stability and a green synthetic method. This strategy follows an approach originally proposed by Barbarella et al. who demonstrated that oxidation of sulfur atoms in oligothiophenes turns them from a *p*-type semiconductor into an *n*-type semiconductor.^84^

TAPPs were also investigated as donors in bulk heterojunction solar cells. Strongly polarized dye **18** was measured in a blend with C70. The performance however was rather poor mostly due to the overall low charge mobility measured for holes (10^–9^ cm^2^/(V s)).^41^ Subsequently, the more complex TAPP **20** (Figure 2) was investigated as a photosensitizer in DSSCs.^43^ The quadrupolar TAPP **21** was prepared as a nonfullerene acceptor in bulk heterojunction organic solar cells. It was revealed that dye **21** has a high stability, adequate energy level, high carrier mobility, good solubility and film formation, which makes it a potential acceptor material in organic solar cell devices.^85^ In 2016, TAPPs were successfully investigated as a light-emitting layer in OLEDs.^86^

**Figure 10:**
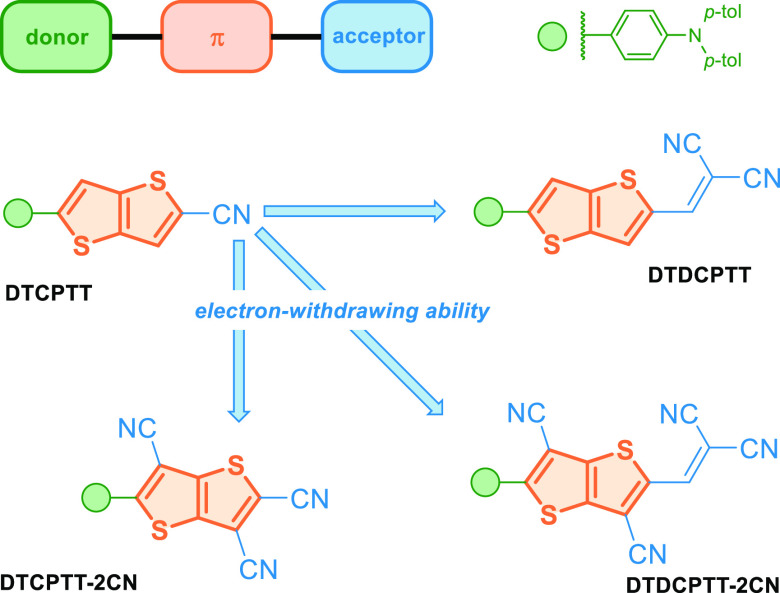
Moderation of an electron-withdrawing nature of acceptor unit in D-π–*A* compounds reported by Wang and co-workers.^87^

A new type of heteropentalenes’-based functional dyes bearing TT core have been reported by Wong and co-workers.^87^ D−π–*A*–type molecules DTCPTT, DTCPTT-2CN, DTDCPTT, and DTDCPTT-2CN exhibit strong ICT absorption in the visible light region (Figure 10). The combined effect of increasing the electron-withdrawing ability of the central (TT to TT-2CN) and terminal end group (CN to DCV) led to bathochromic shift in ICT absorption maxima and significantly lowered LUMO energy levels. Due to their preferred characteristics, namely, high decomposition temperatures together with the appropriate energy level alignment with C_70_, DTDCPTT and DTDCPTT-2CN were chosen as electron donors for vacuum-deposited OPVs. The study has revealed that DTDCPTT- and DTDCPTT-2CN-based devices exhibit excellent indoor photovoltaic (IPV) characteristics. Under 500 lx of TLD-840 fluorescent lamp illumination, the DTDCPTT-based device exhibits the highest PCE, up to 16.9%. Of note, the DTDCPTT-based device is particularly photostable, retaining about 90% of the initial PCE after 465 h, indicating prospects for both efficient and stable IPVs.

**Figure 11:**
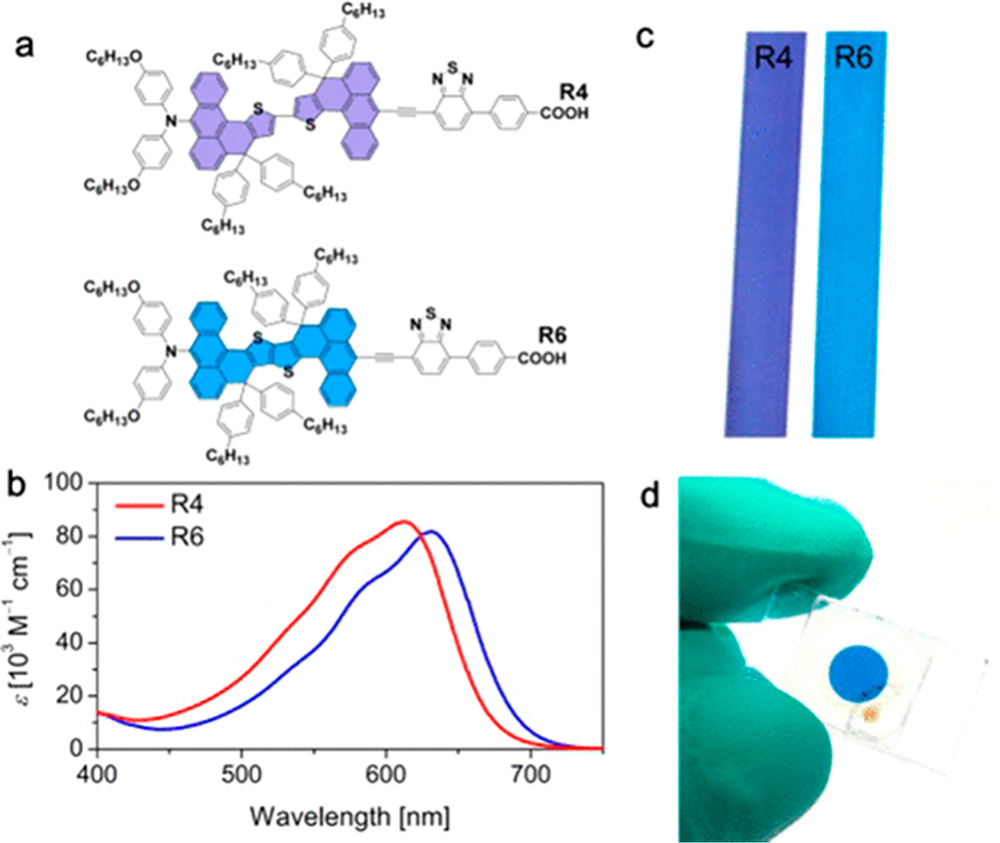
Color palette of photosensitizers achieved by molecular tailoring. (a) Chemical structures of R4 and R6. (b) Spectra of dye molecules in THF (10 μM). (c) Images of dye-sensitized TiO_2_ films (size: 8.5 cm × 1.3 cm). (d) Image of a DSC based on the R6-sensitized nanocrystalline TiO_2_ film with a Co(II/III) tris(bipyridyl)-based electrolyte. Reproduced from ref (88). Copyright 2018 American Chemical Society.

Recently, joint groups of Wang and Grätzel^88^ reported a stable blue polyaromatic hydrocarbon dye R6 involving TT-based core (Figure 11). This dye displayed a brilliant sapphire color in a sensitized TiO_2_ mesoporous film with Co^II/III^/tris(bipyridyl)-based redox electrolyte. As authors disclosed, the R6-based dye-sensitized solar cell (DSC) showed an extraordinary power conversion efficiency (PCE) of 12.6% under full sunlight, which is so far the best performance for all blue DCS dyes. Since the R6-based cell displayed high photostability, this material can be regarded as a promising dye in the DCS color palette with potent application in sunroofs and BIPV windows. Notably, the close analogue of R6, based on the bithiophene core (R4), has a purple color and displays a PCE of 11.1%.

The chemical structure is a key factor that determines the optoelectronic properties of organic electronic materials including heteropentalene-based ones. There is however another important factor which has to be considered, the crystal packing.^89^ In contrast to a solution, where molecules of organic electronics are surrounded by solvent molecules, in the solid state the molecules are close to each other, resulting in a direct overlap of the molecular orbitals of neighboring molecules via moderately weak noncovalent interactions such as hydrogen bonds, π–π stacking, and van der Waals forces, among others.^90^ The formation of different aggregates and arrangements is strongly dependent on the individual molecular structures.^89^ In general, coplanar conjugated molecular structures with effectively extended π-conjugation are favorable for condensed molecular packing which translates to strong intermolecular electronic couplings and large transfer integrals. Not surprisingly, not only an efficient carrier mobility but also a very weak emission efficiency are observed for such dyes. An opposite strategy is beneficial for strong fluorescence. Indeed, in the case of non-coplanar conjugated molecules with bulky substituents and rotating ability, the intermolecular distances become large, resulting in weak intermolecular interactions. Unfortunately, this scenario is unfavorable for efficient charge transport in the solid state, thus giving a rather low carrier mobility.

**Figure 12:**
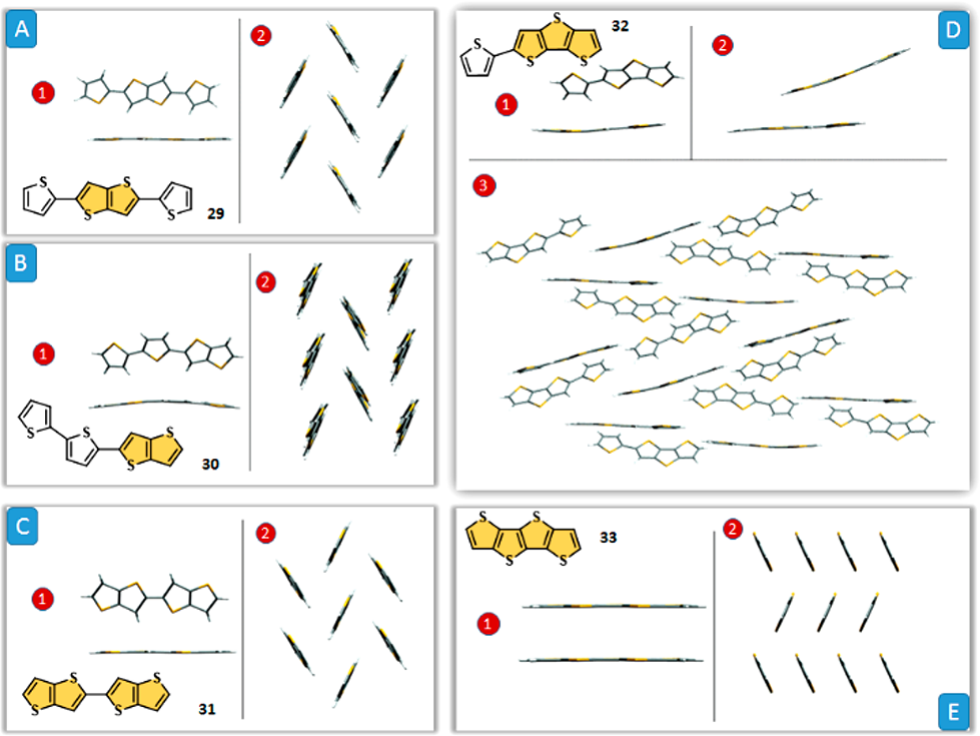
(A–C) Herringbone packed dimer (1) and packing motif (2) of compounds **29**–**31**. (D) Herringbone packed dimer (1), slipped π-stacked dimer (2), and packing motif (3) of compound **32**. (E) π-Stacked dimer and packing motif (2) of compound **33**. Reproduced from ref (91). Copyright 2006 American Chemical Society.

Matzger and co-workers^91^ investigated these phenomena in detail by an inspection of X-ray diffraction of TT-based oligomers **29**–**33** (Figure 12) along with TDDFT calculations probing the electronic transitions of isolated and closely interacting molecules. It was found that the herringbone packing of **29**–**31** causes the formation of H-aggregates and leads to a corresponding blue-shift in the solid-state absorption spectra (Figure 12A–C). The same aggregation type can be also found for other benzo-fused TTs, such as BTBT, DNTT, and DATT (see Figures 3 and 5).^15d,92^ For oligomer **32**, the solid-state spectrum is broadened and contains red-shifted features because the interacting molecules form both H- and J-aggregates in the solid state caused by herringbone and slipped π–π interactions, respectively (Figure 12D). The fully fused oligomer **33** displays a relatively small blue-shift in its solid-state spectrum and adopts a π-stacked packing motif (Figure 12E). The ability to form the different H-, J- and X-aggregate arrangements results in displaying various electrical and optical properties depending on the exciton and splitting energy of such materials.^89^ The hypsochromic shift of absorbance in the solid state along with a low radiative constant (*k*_r_) (with the respect to solution) is typical for H-aggregates. On the other hand, J-aggregate absorbance exhibits a red-shift (bathochromic) and a high *k*_r_. As a result, the formation of H-aggregates often causes quenching in the solid state due to the strong π-overlap, whereas for J-aggregate or herringbone packing the π-overlap decreases and thus Φ_fl_ increases.^15d,90^ A valuable strategy to achieve good mobility and strong emission in crystalline state is either to combine these two modes of assembly (i.e., J-aggregate and a herringbone packing) or to use the X-aggregates which reduces the π-overlap but maintains the planarity.^15d,90^

The intense emission of TAPPs suggests another space for their exploration in fluorescence imaging. The quaternization of DHPPs possessing pyridyl substituents at positions 2 and 5 afforded the double quaternary salts, possessing reasonable water solubility and moderately intense green fluorescence in polar solvents and PBS buffer. These dyes penetrate the membrane of HeLa cells and localize inside in a nonspecific manner. Further experiments have proven that bright fluorescent images can also be obtained under two-photon excitation at 760 nm.^93^

## Summary and Outlook

In conclusion, the rise of optoelectronics has turned scientists’ attention toward heteropentalenes. The combination of superb physicochemical features and straightforward synthesis has contributed to the increase in their popularity in various research applications, as it makes them an ideal platform for many photonics-oriented challenges of modern technology-driven society. The generality of synthetic approaches combined with stability and/or electron-richness has made thieno[3,2-*b*]thiophenes and 1,4-dihydropyrrolo[3,2-*b*]pyrroles the workhorses of this area.

From a preparative point of view, the following strategies prevail: (a) Simultaneous formation of [3,2-*b*]-fused heterocyclic core in π-expanded architectures; (b) Hemetsberger–Knittel reaction; (c) multicomponent condensation leading to tetraaryl-1,4-dihydropyrrolo[3,2-*b*]pyrroles. Future breakthroughs in this aspect are expected to simplify the synthetic access to larger, fused heteropentalenes.

Although the progress in molecular design, engineering, and processing of heteropentalene-based small molecule organic semiconductors (OSCs) has been tremendous, only a selected few have reached field-effect mobilities higher than 10 cm^2^/(V s), typically with single-crystal devices. The research focusing on benzothieno[3,2-*b*]benzothiophenes has demonstrated that they have appropriate HOMO spatial distributions for effective intramolecular overlap and hence lead to OFETs with high mobilities. As far as OFET materials are concerned, in comparison with derivatives of pentacene or rubrene (which holds the record of the highest carrier mobility 20–40 cm^2^/(V s)), π-expanded thieno[3,2-*b*]thiophenes are slightly less effective. The pivotal factor however is that, in contrast to acenes, the chemical stability of HPs is excellent. Their carrier mobility is comparable with that of polycrystalline tetrathiafulvalene, larger than that of semiconducting polymers,^94^ and much larger than that of regioisomeric thieno[3,4-*b*]thiophenes.^2^ They also favorably compare with competing materials as far as the ON/OFF voltage is concerned.

We envision that the next stage of heteropentalene development will be focused mostly on organic field-effect transistors and organic light-emitting diodes. Indeed, applications such as flat-panel display back panels and sensor arrays provide ample motivation to focus on organic semiconductors with higher carrier mobility and better transparency. However, a lot of research is still required to reach this goal. As far as OFETs are concerned, the research will focus on achieving breakthroughs in the enhancement of mobility and improving the environmental robustness. The exploration of HPs possessing two SO_2_ units, as well as based on selenophenes, point toward a new direction in the area, especially benzo[*b*]benzo[4,5]thieno[2,3-*d*]thiophene-5,5,10,10-tetraoxides, which combine stability, large fluorescence quantum yields, and large Stokes shifts. Another important direction is in-depth analysis of factors such as the exciton binding energies, defect states, charge carrier mobilities, and excited state properties, which are pivotal for the device functions. In spite of the fact that many D–A combinations have been reported in recent years for various OPV-related applications, the potential of the DHPP as the most electron-rich small aromatic heterocycle has not been yet fully realized. This will be, in our opinion, one of the most promising directions of future research. Although thus far the exploration of π-expanded heteropentalenes was almost exclusively driven by synthetic accessibility, this picture will be plausibly very different in the near future. The combined effects of reaching synthetic maturity, advances in computational methods, and strong driving force behind organic optoelectronics will favor the theory-driven research activities.

The extensive exploration of [3,2-*b*]-type heteropentalenes has made a broad impact surpassing the applied research related to optoelectronics. They have also contributed, for example, to the understanding of ES-SB and the rise in popularity of fluorescence of nitro-aromatics as a research topic. In the near future, both structures and applications of heteropentalenes will be limited only by our imagination.

## Supporting Information Available

Computational methodologies and Cartesian coordinates (PDF)

## Abbreviations

DHPP: 1,4-dihydropyrrolo[3,2-*b*]pyrrole
TT: thieno[3,2-*b*]thiophene
TAPP: tetraaryl-1,4-dihydropyrrolo[3,2-*b*]pyrrole
*J*_sc_: circuit current density
*V*_oc_: open circuit voltage;

## References

[ref1] aMishra A.; Ma C.-Q.; Bäuerle P. Functional Oligothiophenes: Molecular Design for Multidimensional Nanoarchitectures and Their Applications. Chem. Rev. 2009, 109 (3), 1141–1276. 10.1021/cr8004229. 19209939

[ref2] Zhang C.; Zhu X. Thieno[3,4-b]thiophene-Based Novel Small-Molecule Optoelectronic Materials. Acc. Chem. Res. 2017, 50 (6), 1342–1350. 10.1021/acs.accounts.7b00050. 28375613

[ref3] Janiga A.; Gryko D. T. 1,4-Dihydropyrrolo[3,2-b]pyrrole and Its π-Expanded Analogues. Chem.—Asian J. 2014, 9 (11), 3036–3045. 10.1002/asia.201402367. 24990573

[ref4] Fukazawa A.; Yamaguchi S. Ladder π-Conjugated Materials Containing Main-Group Elements. Chem.—Asian J. 2009, 4 (9), 1386–1400. 10.1002/asia.200900179. 19623599

[ref5] Vogt A.; Henne F.; Wetzel C.; Mena-Osteritz E.; Bäuerle P. Synthesis and characterization of *S,N*-heterotetracenes. Beilstein J. Org. Chem. 2020, 16, 2636–2644. 10.3762/bjoc.16.214. 33178354 PMC7607432

[ref6] aYoun J.; Huang P.-Y.; Huang Y.-W.; Chen M.-C.; Lin Y.-J.; Huang H.; Ortiz R. P.; Stern C.; Chung M.-C.; Feng C.-Y.; Chen L.-H.; Facchetti A.; Marks T. J. Versatile α,ω-Disubstituted Tetrathienoacene Semiconductors for High Performance Organic Thin-Film Transistors. Adv. Funct. Mater. 2012, 22 (1), 48–60. 10.1002/adfm.201101053.

[ref7] Stanforth S. P. Five-five-heteroacenes with one heteroatom in each ring. In Science of Synthesis Knowledge Updates; Banert K., Aitken R. A., Ameduri B., Braverman S., Cherkinsky M., Eds.; Thieme: 2014; Vol. 3.

[ref8] Hemetsberger H.; Knittel D. Synthese und thermolyse von α-azidoacrylestern. Monatsh. Chem. 1972, 103, 194–204. 10.1007/BF00912944.

[ref9] aBulumulla C.; Kularatne R. N.; Gunawardhana R.; Nguyen H. Q.; McCandless G. T.; Biewer M. C.; Stefan M. C. Incorporation of Thieno[3,2-*b*]pyrrole into Diketopyrrolopyrrole-Based Copolymers for Efficient Organic Field Effect Transistors. ACS Macro Lett. 2018, 7 (6), 629–634. 10.1021/acsmacrolett.8b00236. 35632968

[ref10] Stokes B. J.; Dong H.; Leslie B. E.; Pumphrey A. L.; Driver T. G. Intramolecular C–H Amination Reactions: Exploitation of the Rh(II)-Catalyzed Decomposition of Azidoacrylates. J. Am. Chem. Soc. 2007, 129 (24), 7500–7501. 10.1021/ja072219k. 17523647

[ref11] Matteson D. S.; Snyder H. R. A Practical Synthesis of Thieno[3,2-*b*]pyrrole. J. Org. Chem. 1957, 22 (11), 1500–1504. 10.1021/jo01362a048.

[ref12] Henssler J. T.; Matzger A. J. Facile and Scalable Synthesis of the Fused-Ring Heterocycles Thieno[3,2-*b*]thiophene and Thieno[3,2-*b*]furan. Org. Lett. 2009, 11 (14), 3144–3147. 10.1021/ol9010745. 19548691

[ref13] Owczarczyk Z. R.; Braunecker W. A.; Garcia A.; Larsen R.; Nardes A. M.; Kopidakis N.; Ginley D. S.; Olson D. C. 5,10-Dihydroindolo[3,2-*b*]indole-Based Copolymers with Alternating Donor and Acceptor Moieties for Organic Photovoltaics. Macromolecules 2013, 46 (4), 1350–1360. 10.1021/ma301987p.

[ref14] aChen D.; Yuan D.; Zhang C.; Wu H.; Zhang J.; Li B.; Zhu X. Ullmann-Type Intramolecular C–O Reaction Toward Thieno[3,2-*b*]furan Derivatives with up to Six Fused Rings. J. Org. Chem. 2017, 82 (20), 10920–10927. 10.1021/acs.joc.7b01745. 28914541

[ref15] aShan X.-H.; Yang B.; Qu J.-P.; Kang Y.-B. CuSO_4_-Catalyzed dual annulation to synthesize O, S or N-containing tetracyclic heteroacenes. Chem. Commun. 2020, 56 (29), 4063–4066. 10.1039/D0CC01172J. 32162642

[ref16] Hung T. Q.; Hancker S.; Villinger A.; Lochbrunner S.; Dang T. T.; Friedrich A.; Breitsprecher W.; Langer P. Novel synthesis of 5-methyl-5,10-dihydroindolo[3,2-*b*]indoles by Pd-catalyzed C–C and two-fold C–N coupling reactions. Org. Biomol. Chem. 2015, 13 (2), 583–591. 10.1039/C4OB01723D. 25381935

[ref17] Janiga A.; Glodkowska-Mrowka E.; Stoklosa T.; Gryko D. T. Synthesis and Optical Properties of Tetraaryl-1,4-dihydropyrrolo[3,2-*b*]pyrroles. Asian J. Org. Chem. 2013, 2 (5), 411–415. 10.1002/ajoc.201200201.

[ref18] Tasior M.; Vakuliuk O.; Koga D.; Koszarna B.; Górski K.; Grzybowski M.; Kielesiński Ł.; Krzeszewski M.; Gryko D. T. Method for the Large-Scale Synthesis of Multifunctional 1,4-Dihydro-pyrrolo[3,2-*b*]pyrroles. J. Org. Chem. 2020, 85 (21), 13529–13543. 10.1021/acs.joc.0c01665. 32907329 PMC7656515

[ref19] Krzeszewski M.; Tasior M.; Grzybowski M.; Gryko D. T. Synthesis of Tetraaryl-, Pentaaryl-, and Hexaaryl-1,4-dihydropyrrolo[3,2-*b*]pyrroles. Org. Synth. 2021, 98, 242–262. 10.15227/orgsyn.098.0242. 24655027

[ref20] aKrzeszewski M.; Kodama T.; Espinoza E. M.; Vullev V. I.; Kubo T.; Gryko D. T. Nonplanar Butterfly-Shaped π-Expanded Pyrrolopyrroles. Chem.—Eur. J. 2016, 22 (46), 16478–16488. 10.1002/chem.201603282. 27659591

[ref21] Krzeszewski M.; Dobrzycki Ł.; Sobolewski A. L.; Cyrański M. K.; Gryko D. T. Bowl-Shaped Pentagon- and Heptagon-Embedded Nanographene Containing a Central Pyrrolo[3,2-*b*]pyrrole Core. Angew. Chem., Int. Ed. 2021, 60 (27), 14998–15005. 10.1002/anie.202104092. 33831270

[ref22] aTasior M.; Clermont G.; Blanchard-Desce M.; Jacquemin D.; Gryko D. T. Synthesis of Bis(arylethynyl)pyrrolo[3,2-*b*]pyrroles and Effect of Intramolecular Charge Transfer on Their Photophysical Behavior. Chem.—Eur. J. 2019, 25 (2), 598–608. 10.1002/chem.201804325. 30276900

[ref23] aKumagai T.; Tanaka S.; Mukai T. Synthesis of 1,4-dihydropyrrolo[3,2-*b*]pyrrole. Tetrahedron Lett. 1984, 25 (49), 5669–5672. 10.1016/S0040-4039(01)91408-X.

[ref24] Tang S.; Zhang J. Design of donors with broad absorption regions and suitable frontier molecular orbitals to match typical acceptors via substitution on oligo(thienylenevinylene) toward solar cells. J. Comput. Chem. 2012, 33 (15), 1353–1363. 10.1002/jcc.22966. 22488353

[ref25] Matsumura M.; Muranaka A.; Kurihara R.; Kanai M.; Yoshida K.; Kakusawa N.; Hashizume D.; Uchiyama M.; Yasuike S. General synthesis, structure, and optical properties of benzothiophene-fused benzoheteroles containing Group 15 and 16 elements. Tetrahedron 2016, 72 (49), 8085–8090. 10.1016/j.tet.2016.10.048.

[ref26] The HOMO–LUMO energies were calculated by the authors by using the Gaussian quantum mechanical package (for details, see the Supporting Information).

[ref27] Soth S.; Farnierz M.; Paulmier C. Recherches en série hétérocyclique. XXIX. Sur des voies d’accès à des thiéno, sélénolo, furo et pyrrolopyrroles. Can. J. Chem. 1978, 56 (10), 1429–1434. 10.1139/v78-234.

[ref28] aKim B.-G.; Jeong E. J.; Chung J. W.; Seo S.; Koo B.; Kim J. A molecular design principle of lyotropic liquid-crystalline conjugated polymers with directed alignment capability for plastic electronics. Nat. Mater. 2013, 12 (7), 659–664. 10.1038/nmat3595. 23524374

[ref29] aHendriks K. H.; Li W.; Wienk M. M.; Janssen R. A. J. Small-Bandgap Semiconducting Polymers with High Near-Infrared Photoresponse. J. Am. Chem. Soc. 2014, 136 (34), 12130–12136. 10.1021/ja506265h. 25101518

[ref30] Mattiello S.; Sanzone A.; Bruni F.; Gandini M.; Pinchetti V.; Monguzzi A.; Facchinetti I.; Ruffo R.; Meinardi F.; Mattioli G.; Sassi M.; Brovelli S.; Beverina L. Chemically Sustainable Large Stokes Shift Derivatives for High-Performance Large-Area Transparent Luminescent Solar Concentrators. Joule 2020, 4 (9), 1988–2003. 10.1016/j.joule.2020.08.006.

[ref31] Zhou Y.; Zhang M.; Ye J.; Liu H.; Wang K.; Yuan Y.; Du Y.-Q.; Zhang C.; Zheng C.-J.; Zhang X.-H. Efficient solution-processed red organic light-emitting diode based on an electron-donating building block of pyrrolo[3,2-*b*]pyrrole. Org. Electronics 2019, 65, 110–115. 10.1016/j.orgel.2018.11.007.

[ref32] Qiu L.; Yu C.; Zhao N.; Chen W.; Guo Y.; Wan X.; Yang R.; Liu Y. An expedient synthesis of fused heteroacenes bearing a pyrrolo[3,2-*b*]pyrrole core. Chem. Commun. 2012, 48 (100), 12225–12227. 10.1039/c2cc36689d. 23152957

[ref33] Vyas V. S.; Gutzler R.; Nuss J.; Kern K.; Lotsch B. V. Optical gap in herringbone and π-stacked crystals of [1]benzothieno[3,2-*b*]benzothiophene and its brominated derivative. CrystEngComm 2014, 16 (32), 7389–7392. 10.1039/C4CE00752B.

[ref34] Kawabata K.; Usui S.; Takimiya K. Synthesis of Soluble Dinaphtho[2,3-*b*:2′,3′-*f*]thieno[3,2-*b*]thiophene (DNTT) Derivatives: One-Step Functionalization of 2-Bromo-DNTT. J. Org. Chem. 2020, 85 (1), 195–206. 10.1021/acs.joc.9b02585. 31762281

[ref35] Poronik Y. M.; Baryshnikov G. V.; Deperasińska I.; Espinoza E. M.; Clark J. A.; Ågren H.; Gryko D. T.; Vullev V. I. Deciphering the unusual fluorescence in weakly coupled bis-nitro-pyrrolo[3,2-*b*]pyrroles. Commun. Chem. 2020, 3 (1), 190 10.1038/s42004-020-00434-6. PMC9814504 36703353

[ref36] Łukasiewicz Ł. G.; Ryu H. G.; Mikhaylov A.; Azarias C.; Banasiewicz M.; Kozankiewicz B.; Ahn K. H.; Jacquemin D.; Rebane A.; Gryko D. T. Symmetry Breaking in Pyrrolo[3,2-b]pyrroles: Synthesis, Solvatofluorochromism and Two-photon Absorption. Chem.—Asian J. 2017, 12 (14), 1736–1748. 10.1002/asia.201700159. 28398672

[ref37] Stężycki R.; Grzybowski M.; Clermont G.; Blanchard-Desce M.; Gryko D. T. Z-Shaped Pyrrolo[3,2-*b*]pyrroles and Their Transformation into π-Expanded Indolo[3,2-*b*]indoles. Chem.—Eur. J. 2016, 22 (15), 5198–5203. 10.1002/chem.201505052. 26889746

[ref38] Tasior M.; Gryko D. T. Synthesis and Properties of Ladder-Type BN-Heteroacenes and Diazabenzoindoles Built on a Pyrrolopyrrole Scaffold. J. Org. Chem. 2016, 81 (15), 6580–6586. 10.1021/acs.joc.6b01209. 27429058

[ref39] Janiga A.; Krzeszewski M.; Gryko D. T. Diindolo[2,3-*b*:2′,3′-*f*]pyrrolo[3,2-*b*]pyrroles as Electron-Rich, Ladder-Type Fluorophores: Synthesis and Optical Properties. Chem.—Asian J. 2015, 10 (1), 212–218. 10.1002/asia.201402925. 25273980

[ref40] Wu D.; Zheng J.; Xu C.; Kang D.; Hong W.; Duan Z.; Mathey F. Phosphindole fused pyrrolo[3,2-*b*]pyrroles: a new single-molecule junction for charge transport. Dalton Trans 2019, 48 (19), 6347–6352. 10.1039/C9DT01299K. 30994138

[ref41] Domínguez R.; Montcada N. F.; de la Cruz P.; Palomares E.; Langa F. Pyrrolo[3,2-b]pyrrole as the Central Core of the Electron Donor for Solution-Processed Organic Solar Cells. ChemPlusChem. 2017, 82 (7), 1096–1104. 10.1002/cplu.201700158. 31961618

[ref42] Li K.; Liu Y.; Li Y.; Feng Q.; Hou H.; Tang B. Z. 2,5-bis(4-alkoxycarbonylphenyl)-1,4-diaryl-1,4-dihydropyrrolo[3,2-*b*]pyrrole (AAPP) AIEgens: tunable RIR and TICT characteristics and their multifunctional applications. Chem. Sci. 2017, 8 (10), 7258–7267. 10.1039/C7SC03076B. 29081958 PMC5633666

[ref43] Wang J.; Chai Z.; Liu S.; Fang M.; Chang K.; Han M.; Hong L.; Han H.; Li Q.; Li Z. Organic Dyes based on Tetraaryl-1,4-dihydropyrrolo-[3,2-*b*]pyrroles for Photovoltaic and Photocatalysis Applications with the Suppressed Electron Recombination. Chem.—Eur. J. 2018, 24 (68), 18032–18042. 10.1002/chem.201803688. 30307090

[ref44] Jung K. H.; Kim K. H.; Lee D. H.; Jung D. S.; Park C. E.; Choi D. H. Liquid crystalline dialkyl-substituted thienylethenyl [1] benzothieno[3,2-*b*] benzothiophene derivatives for organic thin film transistors. Org. Electronics 2010, 11 (9), 1584–1593. 10.1016/j.orgel.2010.07.008.

[ref45] Friese D. H.; Mikhaylov A.; Krzeszewski M.; Poronik Y. M.; Rebane A.; Ruud K.; Gryko D. T. Pyrrolo[3,2-*b*]pyrroles - From Unprecedented Solvatofluorochromism to Two-Photon Absorption. Chem.—Eur. J. 2015, 21 (50), 18364–18374. 10.1002/chem.201502762. 26511232 PMC4713190

[ref46] Poronik Y. M.; Sadowski B.; Szychta K.; Quina F. H.; Vullev V. I.; Gryko D. T. Revisiting the non-fluorescence of nitroaromatics: presumption versus reality. J. Mater. Chem. C 2022, 10 (8), 2870–2904. 10.1039/D1TC05423F.

[ref47] Dereka B.; Rosspeintner A.; Krzeszewski M.; Gryko D. T.; Vauthey E. Symmetry-Breaking Charge Transfer and Hydrogen Bonding: Toward Asymmetrical Photochemistry. Angew. Chem., Int. Ed. 2016, 55 (50), 15624–15628. 10.1002/anie.201608567. 27862802

[ref48] Ivanov A. I. Theory of Vibrational Spectra of Excited Quadrupolar Molecules with Broken Symmetry. J. Phys. Chem. C 2018, 122 (51), 29165–29172. 10.1021/acs.jpcc.8b10985.

[ref49] Ji Y.; Peng Z.; Tong B.; Shi J.; Zhi J.; Dong Y. Polymorphism-dependent aggregation-induced emission of pyrrolopyrrole-based derivative and its multi-stimuli response behaviors. Dyes Pig 2017, 139, 664–671. 10.1016/j.dyepig.2016.12.061.

[ref50] aSrinivasan S.; Schuster G. B. A Conjoined Thienopyrrole Oligomer Formed by Using DNA as a Molecular Guide. Org. Lett. 2008, 10 (17), 3657–3660. 10.1021/ol801137t. 18686966

[ref51] aKlauk H. Organic Electronics: Materials, Manufacturing, and Applications; Wiley: 2006.

[ref52] Yu L.; Chen M.; Dalton L. R. Ladder polymers: recent developments in syntheses, characterization, and potential applications as electronic and optical materials. Chem. Mater. 1990, 2 (6), 649–659. 10.1021/cm00012a013.

[ref53] De Melo C. P.; Silbey R. Non-linear polamzabilities of conjugated chains: regular polyenes, solitons, and polarons. Chem. Phys. Lett. 1987, 140 (5), 537–541. 10.1016/0009-2614(87)80482-7.

[ref54] aHu W.; Zhu N.; Tang W.; Zhao D. Oligo(p-phenyleneethynylene)s with Hydrogen-Bonded Coplanar Conformation. Org. Lett. 2008, 10 (13), 2669–2672. 10.1021/ol800753z. 18507389

[ref55] aYu Z.-D.; Lu Y.; Wang J.-Y.; Pei J. Conformation Control of Conjugated Polymers. Chem.—Eur. J. 2020, 26 (69), 16194–16205. 10.1002/chem.202000220. 32346938

[ref56] McEntee G. J.; Skabara P. J.; Vilela F.; Tierney S.; Samuel I. D. W.; Gambino S.; Coles S. J.; Hursthouse M. B.; Harrington R. W.; Clegg W. Synthesis and Electropolymerization of Hexadecyl Functionalized Bithiophene and Thieno[3,2-*b*]thiophene End-Capped with EDOT and EDTT Units. Chem. Mater. 2010, 22 (9), 3000–3008. 10.1021/cm100514r.

[ref57] Turbiez M.; Hergué N.; Leriche P.; Frère P. Rigid oligomers based on the combination of 3,6-dimethoxythieno[3,2-*b*]thiophene and 3,4-ethylenedioxythiophene. Tetrahedron Lett. 2009, 50 (51), 7148–7151. 10.1016/j.tetlet.2009.10.021.

[ref58] McEntee G. J.; Vilela F.; Skabara P. J.; Anthopoulos T. D.; Labram J. G.; Tierney S.; Harrington R. W.; Clegg W. Self-assembly and charge transport properties of a benzobisthiazole end-capped with dihexyl thienothiophene units. J. Mater. Chem. 2011, 21 (7), 2091–2097. 10.1039/C0JM02607G.

[ref59] Huang J.; Yu G. Recent progress in quinoidal semiconducting polymers: structural evolution and insight. Mater. Chem. Front. 2021, 5 (1), 76–96. 10.1039/D0QM00509F.

[ref60] Huang J.; Lu S.; Chen P.-A.; Wang K.; Hu Y.; Liang Y.; Wang M.; Reichmanis E. Rational Design of a Narrow-Bandgap Conjugated Polymer Using the Quinoidal Thieno[3,2-*b*]thiophene-Based Building Block for Organic Field-Effect Transistor Applications. Macromolecules 2019, 52 (12), 4749–4756. 10.1021/acs.macromol.9b00370.

[ref61] aLee J.; Rajeeva B. B.; Yuan T.; Guo Z.-H.; Lin Y.-H.; Al-Hashimi M.; Zheng Y.; Fang L. Thermodynamic synthesis of solution processable ladder polymers. Chem. Sci. 2016, 7 (2), 881–889. 10.1039/C5SC02385H. 28791119 PMC5530004

[ref62] aTour J. M.; Lamba J. J. S. Synthesis of planar poly(*p*-phenylene) derivatives for maximization of extended p-conjugation. J. Am. Chem. Soc. 1993, 115 (11), 4935–4936. 10.1021/ja00064a083.

[ref63] Li Y.; Qian D.; Zhong L.; Lin J.-D.; Jiang Z.-Q.; Zhang Z.-G.; Zhang Z.; Li Y.; Liao L.-S.; Zhang F. A fused-ring based electron acceptor for efficient non-fullerene polymer solar cells with small HOMO offset. Nano Energy 2016, 27, 430–438. 10.1016/j.nanoen.2016.07.019.

[ref64] Scherf U.; Muellen K. Poly(arylenes) and poly(arylenevinylenes). 11. A modified two-step route to soluble phenylene-type ladder polymers. Macromolecules 1992, 25 (13), 3546–3548. 10.1021/ma00039a037.

[ref65] Saunthwal R. K.; Danodia A. K.; Saini K. M.; Verma A. K. Ag(I)-Catalyzed cycloisomerization reactions: synthesis of substituted phenanthrenes and naphthothiophenes. Org. Biomol. Chem. 2017, 15 (33), 6934–6942. 10.1039/C7OB01646H. 28786457

[ref66] Jassas R. S.; Mughal E. U.; Sadiq A.; Alsantali R. I.; Al-Rooqi M. M.; Naeem N.; Moussa Z.; Ahmed S. A. Scholl reaction as a powerful tool for the synthesis of nanographenes: a systematic review. RSC Adv. 2021, 11 (51), 32158–32202. 10.1039/D1RA05910F. 35495486 PMC9041733

[ref67] aTakimiya K.; Ebata H.; Sakamoto K.; Izawa T.; Otsubo T.; Kunugi Y. 2,7-Diphenyl[1]benzothieno[3,2-*b*]benzothiophene, A New Organic Semiconductor for Air-Stable Organic Field-Effect Transistors with Mobilities up to 2.0 cm^2^ V^–1^ s^–1^. J. Am. Chem. Soc. 2006, 128 (39), 12604–12605. 10.1021/ja064052l. 17002327

[ref68] Yamamoto T.; Takimiya K. FET Characteristics of Dinaphthothienothiophene (DNTT) on Si/SiO_2_ Substrates with Various Surface-Modifications. J. Photopol. Sci. Technol. 2007, 20 (1), 57–59. 10.2494/photopolymer.20.57.

[ref69] aZschieschang U.; Yamamoto T.; Takimiya K.; Kuwabara H.; Ikeda M.; Sekitani T.; Someya T.; Klauk H. Organic Electronics on Banknotes. Adv. Mater. 2011, 23 (5), 654–658. 10.1002/adma.201003374. 21274915

[ref70] Uno M.; Tominari Y.; Yamagishi M.; Doi I.; Miyazaki E.; Takimiya K.; Takeya J. Moderately anisotropic field-effect mobility in dinaphtho[2,3-*b*:2′,3′-f]thiopheno[3,2-*b*]thiophenes single-crystal transistors. Appl. Phys. Lett. 2009, 94 (22), 223308 10.1063/1.3153119.

[ref71] Nakayama K.; Hirose Y.; Soeda J.; Yoshizumi M.; Uemura T.; Uno M.; Li W.; Kang M. J.; Yamagishi M.; Okada Y.; Miyazaki E.; Nakazawa Y.; Nakao A.; Takimiya K.; Takeya J. Patternable Solution-Crystallized Organic Transistors with High Charge Carrier Mobility. Adv. Mater. 2011, 23 (14), 1626–1629. 10.1002/adma.201004387. 21472790

[ref72] aTsutsui Y.; Schweicher G.; Chattopadhyay B.; Sakurai T.; Arlin J.-B.; Ruzié C.; Aliev A.; Ciesielski A.; Colella S.; Kennedy A. R.; Lemaur V.; Olivier Y.; Hadji R.; Sanguinet L.; Castet F.; Osella S.; Dudenko D.; Beljonne D.; Cornil J.; Samorì P.; Seki S.; Geerts Y. H. Unraveling Unprecedented Charge Carrier Mobility through Structure Property Relationship of Four Isomers of Didodecyl[1]benzothieno[3,2-*b*][1]benzothiophene. Adv. Mater. 2016, 28 (33), 7106–7114. 10.1002/adma.201601285. 27226066

[ref73] Yuan Y.; Giri G.; Ayzner A. L.; Zoombelt A. P.; Mannsfeld S. C. B.; Chen J.; Nordlund D.; Toney M. F.; Huang J.; Bao Z. Ultra-high mobility transparent organic thin film transistors grown by an off-centre spin-coating method. Nat. Commun. 2014, 5 (1), 3005 10.1038/ncomms4005. 24398476

[ref74] aBulumulla C.; Gunawardhana R.; Yoo S. H.; Mills C. R.; Kularatne R. N.; Jackson T. N.; Biewer M. C.; Gomez E. D.; Stefan M. C. The effect of single atom replacement on organic thin film transistors: case of thieno[3,2-*b*]pyrrole vs. furo[3,2-*b*]pyrrole. J. Mater. Chem. C 2018, 6 (37), 10050–10058. 10.1039/C8TC02887G.

[ref75] Patra A.; Bendikov M. Polyselenophenes. J. Mater. Chem. 2010, 20 (3), 422–433. 10.1039/B908983G.

[ref76] Fringuelli F.; Marino G.; Taticchi A.; Grandolini G. A comparative study of the aromatic character of furan, thiophen, selenophen, and tellurophen. J. Chem. Soc., Perkin Trans. 1974, 2 (4), 332–337. 10.1039/p29740000332.

[ref77] Li Y.; Zhong L.; Wu F.-P.; Yuan Y.; Bin H.-J.; Jiang Z.-Q.; Zhang Z.; Zhang Z.-G.; Li Y.; Liao L.-S. Non-fullerene polymer solar cells based on a selenophene-containing fused-ring acceptor with photovoltaic performance of 8.6. Energy Envir. Sci. 2016, 9 (11), 3429–3435. 10.1039/C6EE00315J.

[ref78] Wang J.-L.; Liu K.-K.; Hong L.; Ge G.-Y.; Zhang C.; Hou J. Selenopheno[3,2-*b*]thiophene-Based Narrow-Bandgap Nonfullerene Acceptor Enabling 13.3% Efficiency for Organic Solar Cells with Thickness-Insensitive Feature. ACS Energy Lett. 2018, 3 (12), 2967–2976. 10.1021/acsenergylett.8b01808.

[ref79] Lin F.; Zuo L.; Gao K.; Zhang M.; Jo S. B.; Liu F.; Jen A. K. Y. Regio-Specific Selenium Substitution in Non-Fullerene Acceptors for Efficient Organic Solar Cells. Chem. Mater. 2019, 31 (17), 6770–6778. 10.1021/acs.chemmater.9b01242.

[ref80] Yang C.; An Q.; Bai H.-R.; Zhi H.-F.; Ryu H. S.; Mahmood A.; Zhao X.; Zhang S.; Woo H. Y.; Wang J.-L. A Synergistic Strategy of Manipulating the Number of Selenophene Units and Dissymmetric Central Core of Small Molecular Acceptors Enables Polymer Solar Cells with 17.5% Efficiency. Angew. Chem., Int. Ed. 2021, 60 (35), 19241–19252. 10.1002/anie.202104766. 34051037

[ref81] Kukhta N. A.; Marks A.; Luscombe C. K. Molecular Design Strategies toward Improvement of Charge Injection and Ionic Conduction in Organic Mixed Ionic–Electronic Conductors for Organic Electrochemical Transistors. Chem. Rev. 2022, 122, 4325 10.1021/acs.chemrev.1c00266. 34902244 PMC8874907

[ref82] aGiovannitti A.; Sbircea D.-T.; Inal S.; Nielsen C. B.; Bandiello E.; Hanifi D. A.; Sessolo M.; Malliaras G. G.; McCulloch I.; Rivnay J. Controlling the mode of operation of organic transistors through side-chain engineering. Proc. Natl. Acad. Sci. U.S.A. 2016, 113 (43), 12017–12022. 10.1073/pnas.1608780113. 27790983 PMC5087003

[ref83] aJoly D.; Pellejà L.; Narbey S.; Oswald F.; Chiron J.; Clifford J. N.; Palomares E.; Demadrille R. A Robust Organic Dye for Dye Sensitized Solar Cells Based on Iodine/Iodide Electrolytes Combining High Efficiency and Outstanding Stability. Sci. Rep. 2015, 4 (1), 4033 10.1038/srep04033. PMC3916786 24504344

[ref84] Barbarella G.; Favaretto L.; Sotgiu G.; Antolini L.; Gigli G.; Cingolani R.; Bongini A. Rigid-Core Oligothiophene-S,S-dioxides with High Photoluminescence Efficiencies Both in Solution and in the Solid State. Chem. Mater. 2001, 13 (11), 4112–4122. 10.1021/cm010436t.

[ref85] Zhenglong Y. Synthesis method of poly-thiophene-fullerene-polylactic acid triblock copolymer. CN102391481A, 2012.

[ref86] Hayama T.; Kawamura M.; Mizuki Y.; Ito H.; Haketa T.; Gryko D. T.; Janiga A.; Krzeszewski M. Material for organic electroluminescent element, organic electroluminescent element and electronic apparatus. JP2016127083A, 2016.

[ref87] Su J. M.; Li Y. Z.; Chang Y. H.; Li M. Z.; Qiu W. Z.; Liu S. W.; Wong K. T. Novel thieno[3,2-*b*]thiophene-embedded small-molecule donors for highly efficient and photostable vacuum-processed organic photovoltaics. Mater. Today Energy 2021, 20, 100633 10.1016/j.mtener.2020.100633.

[ref88] Ren Y.; Sun D.; Cao Y.; Tsao H. N.; Yuan Y.; Zakeeruddin S. M.; Wang P.; Grätzel M. A Stable Blue Photosensitizer for Color Palette of Dye-Sensitized Solar Cells Reaching 12.6% Efficiency. J. Am. Chem. Soc. 2018, 140 (7), 2405–2408. 10.1021/jacs.7b12348. 29323883

[ref89] aFang H.-H.; Yang J.; Feng J.; Yamao T.; Hotta S.; Sun H.-B. Functional organic single crystals for solid-state laser applications. Laser Photonics Rev. 2014, 8 (5), 687–715. 10.1002/lpor.201300222.

[ref90] Yu P.; Zhen Y.; Dong H.; Hu W. Crystal Engineering of Organic Optoelectronic Materials. Chem. 2019, 5 (11), 2814–2853. 10.1016/j.chempr.2019.08.019.

[ref91] Zhang X.; Johnson J. P.; Kampf J. W.; Matzger A. J. Ring Fusion Effects on the Solid-State Properties of α-Oligothiophenes. Chem. Mater. 2006, 18 (15), 3470–3476. 10.1021/cm0609348.

[ref92] aKang M. J.; Miyazaki E.; Osaka I.; Takimiya K.; Nakao A. Diphenyl Derivatives of Dinaphtho[2,3-b:2′,3′-f]thieno[3,2-b]thiophene: Organic Semiconductors for Thermally Stable Thin-Film Transistors. ACS Appl. Mater. Interfaces 2013, 5 (7), 2331–2336. 10.1021/am3026163. 23410846

[ref93] Santra M.; Jun Y. W.; Bae J.; Sarkar S.; Choi W.; Gryko D. T.; Ahn K. H. Water-Soluble Pyrrolo[3,2-b]pyrroles: Synthesis, Luminescence and Two-Photon Cellular Imaging Properties. Asian J. Org. Chem. 2017, 6 (3), 278–281. 10.1002/ajoc.201600613.

[ref94] Wu Y.; Zhao Y.; Liu Y. Toward Efficient Charge Transport of Polymer-Based Organic Field-Effect Transistors: Molecular Design, Processing, and Functional Utilization. Acc. Mater. Res. 2021, 2 (11), 1047–1058. 10.1021/accountsmr.1c00149.

