# Supplementary material for: Multifunctional Heteropentalenes: From Synthesis to Optoelectronic Applications
Source: JACS Au. 2022 May 10;2(6):1290–305. doi: 10.1021/jacsau.2c00147 (PMC9241017; doi:10.1021/jacsau.2c00147)
Supplement: Supplementary file 1 — au2c00147_si_001.pdf [file au2c00147_si_001.pdf]

Multifunctional Heteropentalenes: From Synthesis to Optoelectronic Applications

Sebastian Stecko[a]* and Daniel T. Gryko[a]*

[a]Institute of Organic Chemistry, Polish Academy of Sciences, Kasprzaka 44-52, 01-224 Warsaw, Poland

* E-mail: dtgryko@icho.edu.pl

* E-mail: sebastian.stecko@icho.edu.pl

Electronic supporting information

The DFT computation

Geometry optimizations and energies of HOMO-LUMO orbitals were computed via PBE0/6-31(d) method (for heavy atoms (Se and Te atoms SDD was applied) by using The Gaussian 03[1] quantum mechanical package.

References:

[1] Frisch, M. J.; Trucks, G. W.; Schlegel, H. B.; Scuseria, G. E.; Robb, M. A.; Cheeseman, J. R.; Montgomery, Jr., J. A.; Vreven, T.; Kudin, K. N.; Burant, J. C.; Millam, J. M.; Iyengar, S. S.; Tomasi, J.; Barone, V.; Mennucci, B.; Cossi, M.; Scalmani, G.; Rega, N.; Petersson, G. A.; Nakatsuji, H.; Hada, M.; Ehara, M.; Toyota, K.; Fukuda, R.; Hasegawa, J.; Ishida, M.; Nakajima, T.; Honda, Y.; Kitao, O.; Nakai, H.; Klene, M.; Li, X.; Knox, J. E.; Hratchian, H. P.; Cross, J. B.; Bakken, V.; Adamo, C.; Jaramillo, J.; Gomperts, R.; Stratmann, R. E.; Yazyev, O.; Austin, A. J.; Cammi, R.; Pomelli, C.; Ochterski, J. W.; Ayala, P. Y.; Morokuma, K.; Voth, G. A.; Salvador, P.; Dannenberg, J. J.; Zakrzewski, V. G.; Dapprich, S.; Daniels, A. D.; Strain, M. C.; Farkas, O.; Malick, D. K.; Rabuck, A. D.; Raghavachari, K.; Foresman, J. B.; Ortiz, J. V.; Cui, Q.; Baboul, A. G.; Clifford, S.; Cioslowski, J.; Stefanov, B. B.; Liu, G.; Liashenko, A.; Piskorz, P.; Komaromi, I.; Martin, R. L.; Fox, D. J.; Keith, T.; Al-Laham, M. A.; Peng, C. Y.; Nanayakkara, A.; Challacombe, M.; Gill, P. M. W.; Johnson, B.; Chen, W.; Wong, M. W.; Gonzalez, C.; Pople, J. A.; Gaussian 03, revision D.01; Gaussian, Inc.: Wallingford CT, 2004

Cartesian coordinates

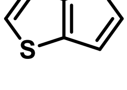

0 1

C	-0.33715100	-2.27630300	0.00004700
C	-1.29810800	-1.29032900	-0.00004000
C	-0.70169200	0.00556300	-0.00010700
C	0.70169200	-0.00556300	-0.00010700
H	-0.48850500	-3.35578800	0.00002100
H	-2.37108800	-1.49153700	-0.00007300
C	1.29810800	1.29032900	-0.00004000
H	2.37108800	1.49153700	-0.00007300

C	0.33715100	2.27630300	0.00004700
H	0.48850500	3.35578800	0.00002100
S	-1.29810800	1.64734400	0.00004100
S	1.29810800	-1.64734400	0.00004100

1 2 2.0 5 1.0 12 1.0
2 3 1.5 6 1.0
3 4 1.5 11 1.0
4 7 1.5 12 1.0
5
6
7 8 1.0 9 2.0
8
9 10 1.0 11 1.0
10
11
12

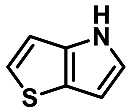

0 1

C	-1.93787500	0.74759900	-0.00001900
C	-0.82573900	1.56128000	-0.00004600
C	0.36515200	0.77675900	-0.00000500
C	0.15605200	-0.61584200	0.00006000
H	-2.98438000	1.05176000	-0.00000900
H	-0.87554100	2.65234700	-0.00008700
C	1.42191500	-1.26778400	0.00004200
H	1.63194000	-2.33571500	0.00005900
C	2.37059700	-0.25173600	-0.00004400
H	3.45773400	-0.31020400	-0.00006000
S	-1.55302700	-0.96801000	0.00006000
N	1.73108900	0.97853800	-0.00011400
H	2.20045100	1.87854100	0.00000700

1 2 2.0 5 1.0 11 1.0
2 3 1.5 6 1.0
3 4 1.5 12 1.5
4 7 1.5 11 1.0
5
6
7 8 1.0 9 1.5
8
9 10 1.0 12 1.0
10
11
12 13 1.0
13

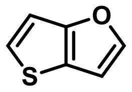

0 1

C	-1.91751400	0.74480600	0.00026700
C	-0.80265700	1.55696200	0.00014300
C	0.36766200	0.75205900	-0.00018600
C	0.16300800	-0.62575500	-0.00028000
H	-2.96363000	1.04986500	0.00023800
H	-0.83383200	2.64731000	0.00019000
C	1.45641500	-1.23853700	-0.00009200
H	1.71200000	-2.29653400	0.00003700
C	2.34265600	-0.18744100	0.00037500
H	3.42914800	-0.13921900	0.00069300
O	1.70616400	1.03944500	-0.00036900
S	-1.54065200	-0.97434700	0.00002700

1 2 2.0 5 1.0 12 1.0
2 3 1.5 6 1.0
3 4 1.5 11 1.0
4 7 1.5 12 1.0
5
6
7 8 1.0 9 2.0
8
9 10 1.0 11 1.0
10
11
12

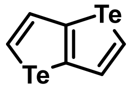

0 1

C	-1.41034000	-2.03064600	-0.00015900
C	-1.74223500	-0.70063000	-0.00004600
C	-0.65689000	0.23373000	0.00023000
C	0.65689000	-0.23373000	0.00023000
H	-2.11071700	-2.86699100	-0.00102500
H	-2.78945200	-0.37992800	0.00005700
C	1.74223500	0.70063000	-0.00004600
H	2.78945200	0.37992800	0.00005700
C	1.41034000	2.03064600	-0.00015900
H	2.11071700	2.86699100	-0.00102500
Te	0.65689000	-2.33949500	0.00001600
Te	-0.65689000	2.33949500	0.00001600

1 2 2.0 5 1.0 11 1.0
2 3 1.5 6 1.0
3 4 1.5 12 1.0
4 7 1.5 11 1.0
5
6
7 8 1.0 9 2.0
8
9 10 1.0 12 1.0
10
11
12

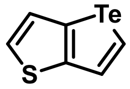

0 1

C	-1.01137800	1.72767800	0.00059300
C	0.34475600	1.91292800	0.00021000
C	1.14551200	0.72308000	0.00002000
C	0.52459300	-0.53239600	0.00007500
H	-1.76522000	2.51583900	0.00008000
H	0.78562100	2.91507900	0.00045800
C	1.43284200	-1.62998800	0.00031900
H	1.14550800	-2.68356400	0.00102900
C	2.74409700	-1.20325400	0.00019400
H	3.64748400	-1.81351400	0.00074700
S	2.88682400	0.53561700	-0.00033900
Te	-1.55932900	-0.29792300	-0.00010300

1 2 2.0 5 1.0 12 1.0
2 3 1.5 6 1.0
3 4 1.5 11 1.0
4 7 1.5 12 1.0
5
6
7 8 1.0 9 2.0
8

9 10 1.0 11 1.0
10
11
12

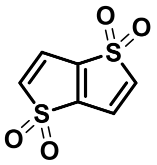

0 1
C -1.59849000 1.75241600 -0.00018000
C -1.82158200 0.41768700 -0.00031900
C -0.58039700 -0.34956900 -0.00060600
C 0.58039700 0.34956900 -0.00060600
H -2.31079000 2.57809100 0.00044900
H -2.81079300 -0.04644200 -0.00056200
C 1.82158200 -0.41768700 -0.00031900
H 2.81079300 0.04644200 -0.00056200
C 1.59849000 -1.75241600 -0.00018000
H 2.31079000 -2.57809100 0.00044900
S 0.17872500 2.11958600 0.00008900
S -0.17872500 -2.11958600 0.00008900
O 0.58006700 2.73873700 -1.28432400
O 0.58039700 2.73738200 1.28498800
O -0.58006700 -2.73873700 -1.28432400
O -0.58039700 -2.73738200 1.28498800

1 2 2.0 5 1.0 11 1.0
2 3 1.0 6 1.0
3 4 2.0 12 1.0
4 7 1.0 11 1.0
5
6
7 8 1.0 9 2.0
8
9 10 1.0 12 1.0
10
11 13 2.0 14 2.0
12 15 2.0 16 2.0
13
14
15
16

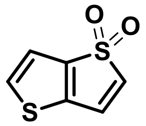

0 1
C 1.36226400 1.57475100 0.00000300
C 0.04896700 1.89946800 -0.00004900
C -0.82108300 0.72552400 -0.00020000
C -0.18391700 -0.50285400 -0.00031200
H 2.23711500 2.22508700 0.00089400
H -0.32873000 2.92537300 -0.00007500
C -1.04873600 -1.62391700 -0.00003600
H -0.73197800 -2.66826500 -0.00019400
C -2.37146100 -1.21763800 0.00002800
H -3.26591400 -1.84077300 0.00056100
S 1.59955200 -0.22145100 0.00003700
O 2.20369300 -0.65765400 -1.28223300
O 2.20327700 -0.65767100 1.28238800
S -2.54220500 0.51827400 0.00002300

1 2 2.0 5 1.0 11 1.0
2 3 1.0 6 1.0
3 4 2.0 14 1.0
4 7 1.5 11 1.0
5
6
7 8 1.0 9 2.0

8
9 10 1.0 14 1.0
10
11 12 2.0 13 2.0
12
13
14

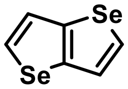

0 1
C -1.25911400 -2.02488900 0.00049100
C -1.70554700 -0.72863300 -0.00013800
C -0.65425200 0.23849400 -0.00084300
C 0.65425200 -0.23849400 -0.00084300
H -1.85421800 -2.93809800 -0.00042100
H -2.76905100 -0.47531300 0.00012100
C 1.70554700 0.72863300 -0.00013800
H 2.76905100 0.47531300 0.00012100
C 1.25911400 2.02488900 0.00049100
H 1.85421800 2.93809800 -0.00042100
Se 0.65425200 -2.15910000 0.00009500
Se -0.65425200 2.15910000 0.00009500

1 2 2.0 5 1.0 11 1.0
2 3 1.5 6 1.0
3 4 1.5 12 1.0
4 7 1.5 11 1.0
5
6
7 8 1.0 9 2.0
8
9 10 1.0 12 1.0
10
11
12

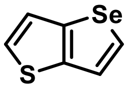

0 1
C -1.49173400 1.45388400 0.00051400
C -0.17640500 1.83532000 -0.00006100
C 0.73902600 0.73476700 -0.00047200
C 0.18683500 -0.55031700 -0.00057400
H -2.37324900 2.09483800 -0.00072900
H 0.13045000 2.88453700 0.00008100
C 1.14309200 -1.60498300 -0.00000700
H 0.90805200 -2.67116000 0.00020300
C 2.42901600 -1.10750300 0.00024100
H 3.36331600 -1.66915400 0.00029200
Se -1.72818500 -0.45404200 0.00003400
S 2.48442200 0.63945900 0.00007300

1 2 2.0 5 1.0 11 1.0
2 3 1.5 6 1.0
3 4 1.5 12 1.0
4 7 1.5 11 1.0
5
6
7 8 1.0 9 2.0
8
9 10 1.0 12 1.0
10
11
12

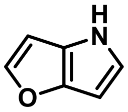

0 1

C	-2.09869000	-0.02095700	0.00022100
C	-1.37010500	1.14869600	0.00019200
C	-0.00661300	0.71472800	-0.00013500
C	-0.01431300	-0.67978700	-0.00024800
H	-3.16854500	-0.21616000	0.00030700
H	-1.78096700	2.15711500	0.00038000
C	1.31271000	-1.18036700	0.00004200
H	1.66338400	-2.21010700	0.00013400
C	2.11281200	-0.03805200	0.00043600
H	3.19780600	0.04845000	0.00074400
N	1.31659200	1.10367900	-0.00020700
H	1.66818700	2.05532100	-0.00097800
O	-1.30135100	-1.15324200	-0.00027400

1 2 2.0 5 1.0 13 1.0
2 3 1.5 6 1.0
3 4 1.5 11 1.5
4 7 1.5 13 1.0
5
6
7 8 1.0 9 1.5
8
9 10 1.0 11 1.0
10
11 12 1.0
12
13

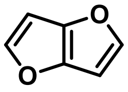

0 1

C	-0.01065200	2.07997600	-0.00027000
C	1.15411100	1.34019300	-0.00002600
C	0.68951600	-0.00725300	-0.00000200
C	-0.68951600	0.00725300	-0.00000200
H	-0.19745000	3.15094300	-0.00068300
H	2.16868100	1.73291700	-0.00011600
C	-1.15411100	-1.34019300	-0.00002600
H	-2.16868100	-1.73291700	-0.00011600
C	0.01065200	-2.07997600	-0.00027000
H	0.19745000	-3.15094300	-0.00068300
O	1.15411100	-1.29206700	0.00032300
O	-1.15411100	1.29206700	0.00032300

1 2 2.0 5 1.0 12 1.0
2 3 1.5 6 1.0
3 4 2.0 11 1.0
4 7 1.5 12 1.0
5
6
7 8 1.0 9 2.0
8
9 10 1.0 11 1.0
10
11
12

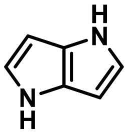

0 1

C	-1.71888400	1.26312200	-0.00035700
C	-0.41021900	1.73833200	-0.00014400
C	0.41021900	0.57276000	0.00062200
C	-0.41021900	-0.57276000	0.00062200
H	-2.65801700	1.81376400	-0.00103200
H	-0.11494500	2.78640100	-0.00037200

C	0.41021900	-1.73833200	-0.00014400
H	0.11494500	-2.78640100	-0.00037200
C	1.71888400	-1.26312200	-0.00035700
H	2.65801700	-1.81376400	-0.00103200
N	1.71900700	0.12726500	0.00017800
H	2.55099300	0.70694500	-0.00056400
N	-1.71900700	-0.12726500	0.00017800
H	-2.55099300	-0.70694500	-0.00056400

1 2 1.5 5 1.0 13 1.0
2 3 1.5 6 1.0
3 4 1.5 11 1.0
4 7 1.5 13 1.0
5
6
7 8 1.0 9 1.5
8
9 10 1.0 11 1.0
10
11 12 1.0
12
13 14 1.0
14
